# Multi-Modal Feature Fusion and Hierarchical Classification for Automated Equine–Human Interaction Behavior Recognition

**DOI:** 10.3390/s26072202

**Published:** 2026-04-02

**Authors:** Samierra Arora, Emily Kieson, Christine Rudd, Peter A. Gloor

**Affiliations:** 1 System Design & Management, Massachusetts Institute of Technology, Cambridge, MA 02142, USA; samierraarora@gmail.com; 2 Equine International, Boston, MA 02115, USA; ekieson@equineintl.org (E.K.); ruddc@equineintl.org (C.R.); 3 Cologne Institute for Information Sciences, University of Cologne, 50923 Cologne, Germany

**Keywords:** equine behavior, human–animal interaction, hierarchical classification, multi-modal feature fusion, class imbalance

## Abstract

Automated recognition of equine–human interaction behaviors from video represents a significant challenge in computational ethology, with critical applications spanning animal welfare assessment, equine-assisted services evaluation, and safety monitoring in equestrian environments. Existing approaches to animal behavior recognition typically focus on single species in isolation, rely solely on facial expression analysis while ignoring full-body posture, or employ flat classification architectures that fail under the severe class imbalances characteristic of naturalistic behavioral datasets. Furthermore, no prior framework integrates simultaneous analysis of both human and equine body language for cross-species interaction classification. This paper presents a novel hierarchical classification framework integrating multi-modal computer vision features to distinguish behavioral states during horse–human encounters. Our methodology employs three complementary feature extraction pipelines: YOLOv8 for spatial relationship modeling, MediaPipe for human postural analysis, and AP-10K for equine body language interpretation. From 28 annotated interaction videos comprising 50,270 temporal samples across five horse breeds, we extract 35 discriminative features capturing proximity dynamics, body orientation, and species-specific behavioral indicators. To address severe class imbalance (18.3:1 ratio between affiliative and avoidant categories), we implement cost-sensitive gradient boosting with automatic class weight optimization within a two-stage hierarchical architecture. The first stage classifies interactions into three parent categories (affiliative, neutral, avoidant) achieving 73.2% balanced accuracy, while stage two discriminates six fine-grained sub-behaviors achieving 88.5% balanced accuracy (under oracle parent-category routing; cascaded end-to-end performance is 62.9% balanced accuracy due to Stage 1 error propagation, identifying parent classification as the primary bottleneck). Notably, our system achieves 85.0% recall on safety-critical avoidant behaviors despite their representation of only 3.8% of the dataset. Extensive ablation studies demonstrate that equine pose features contribute most critically to classification performance, while comprehensive cross-validation analysis confirms model robustness across diverse interaction contexts. The proposed framework establishes the first systematic multimodal cross-species behavioral assessment pipeline in human–animal interaction research, with direct implications for improving equine welfare monitoring and rider safety protocols.

## 1. Introduction

The scientific study of human–animal interactions has emerged as a critical interdisciplinary field with implications spanning animal welfare science, therapeutic intervention design, and occupational safety protocols [1]. Among domesticated species, the horse (*Equus caballus*) presents particularly complex behavioral dynamics due to its evolutionary heritage as a prey animal, sophisticated social cognition, and extensive history of human partnership across diverse contexts including agriculture, sport, therapy, and companionship [2,3].

Accurate interpretation of equine behavioral states during human interaction carries substantial practical and safety significance. The global equine industry represents a $300 billion annual economic sector, with over 60 million horses worldwide engaged in sport, recreation, and therapy programs [4]. Within equine-assisted services contexts, recognizing subtle shifts from affiliative engagement toward stress indicators enables practitioners to modify sessions before behavioral escalation, directly impacting treatment outcomes for participants, including those with autism spectrum disorder [5,6], cerebral palsy [7], and post-traumatic stress disorder [8]. In professional equestrian environments, early detection of avoidant behaviors prevents accidents that result in approximately 20 fatalities and 100,000 emergency department visits annually in the United States alone [9]. Furthermore, objective behavioral assessment tools support evidence-based animal welfare evaluation, replacing subjective observer judgments with quantifiable metrics that can be systematically monitored and improved.

Despite this importance, automated recognition of equine–human interaction behaviors from video remains substantially underexplored compared to human-centric action recognition or single-species animal behavior analysis. The challenge is multifaceted: horses and humans occupy shared spatial environments requiring simultaneous tracking of both subjects; relevant behavioral indicators span both species’ body language with distinct modalities (facial expressions, ear positions, postural configurations); and the temporal dynamics of interaction unfold across variable timescales from momentary gestures to sustained engagement patterns lasting minutes.

Previous computational approaches to equine behavior analysis have primarily focused on isolated horse behaviors such as gait classification for lameness detection [10], pain grimace recognition using facial feature extraction [11,12], or social hierarchy assessment within horse groups through proximity analysis [13]. Studies addressing human–horse dyadic interactions have typically relied on manual annotation by trained ethologists using standardized ethogram protocols [14], limiting scalability to large datasets and introducing inter-observer variability that can exceed 20% even among experienced coders [15].

However, systematic frameworks for integrating these multi-modal signals into interpretable behavioral classifications for cross-species interactions remain underdeveloped. Research on human–animal interaction behavior coding has established foundational ethological frameworks [1,14,16], and recent computational approaches have begun to address individual species’ emotional states [17,18,19]. Yet existing animal behavior recognition systems either focus on single species in isolation, analyze only facial expressions while ignoring full-body posture, or employ flat classification architectures that struggle with the severe class imbalances inherent in naturalistic behavioral datasets where rare but safety-critical categories (e.g., aggressive or avoidant responses) may represent less than 5% of observations.

This paper addresses these gaps through the following primary contributions:

1. **Multi-Modal Feature Extraction Pipeline**: We develop an integrated framework combining YOLOv8 object detection for spatial relationship modeling, MediaPipe for human pose estimation, and AP-10K for equine body language analysis, extracting 35 complementary features that capture proximity, orientation, and species-specific behavioral indicators (see architecture overview in Section 4).
2. **Hierarchical Classification Architecture**: We introduce a two-stage classification system that first discriminates coarse parent categories (affiliative, neutral, avoidant) before performing fine-grained sub-behavior classification within each category, substantially improving recognition of minority classes under severe imbalance (18.3:1 ratio).
3. **Comprehensive Empirical Evaluation**: Through analysis of 50,270 annotated temporal samples from 28 videos spanning five horse breeds and diverse interaction contexts, we establish the first systematic multimodal cross-species interaction framework, achieving competitive performance including 88.5% balanced accuracy on six-behavior classification and 85.0% recall on safety-critical avoidant behaviors, validated through rigorous cross-validation and ablation studies.

The remainder of this paper is organized as follows. Section 2 reviews related work in animal behavior recognition, equine ethology, and multi-modal classification systems. Section 3 details our dataset collection, annotation protocol, and behavioral taxonomy. Section 4 describes the multi-modal feature extraction methodologies. Section 5 presents the hierarchical classification framework and training procedures. Section 6 reports comprehensive experimental results including ablation studies, cross-validation analysis, and comparison with baseline methods. Section 7 discusses implications, limitations, and future research directions. Section 8 concludes with summary remarks and practical recommendations for deployment.

## 2. Related Work

### 2.1. Automated Animal Behavior Recognition

The application of computer vision and machine learning to animal behavior analysis has accelerated dramatically over the past decade. DeepLabCut [20] introduced a transfer-learning approach to animal pose estimation that eliminated the need for physical markers, making naturalistic behavioral studies tractable at scale. Building on this foundation, SLEAP [21] addressed the multi-animal tracking problem by incorporating identity assignment across frames. The AP-10K dataset [22] provided large-scale benchmarks for animal pose estimation across 54 diverse species including horses, establishing pretrained models applicable to equine subjects with transfer learning.

For behavior classification specifically, recurrent neural networks and temporal convolutional networks have demonstrated effectiveness in capturing sequential dynamics [23]. However, these approaches typically require substantial training data volumes (tens of thousands of labeled clips) that may be unavailable for specialized domains such as human–horse interaction. Alternative approaches employing traditional machine learning classifiers (random forests, gradient boosting) on engineered features offer interpretability advantages and reduced data requirements, motivating our methodology.

Recent work in animal behavior recognition has begun addressing class imbalance through cost-sensitive learning [24], synthetic data augmentation via SMOTE and variants [25,26], and hierarchical classification strategies that decompose complex multi-class problems into more tractable binary decisions [27]. These techniques prove particularly relevant for equine welfare applications where rare but critical behaviors (pain indicators, stress responses) must be reliably detected despite representing small fractions of observational data.

### 2.2. Equine Behavior Analysis and Welfare Assessment

Research specifically addressing equine behavior recognition has followed several trajectories. Gait analysis systems classify locomotion patterns (walk, trot, canter, gallop) from video or wearable sensor data, supporting lameness detection and performance optimization [10]. The Equine Pain Face method [11] pioneered systematic scoring of facial expressions indicating discomfort through action units analogous to the Facial Action Coding System (FACS) used in humans. Dalla Costa et al. [12] developed the Horse Grimace Scale (HGS) as a practical tool for pain assessment, subsequently automated through CNN-based classifiers. In parallel, Feighelstein et al. [18] achieved 76% accuracy in distinguishing four equine emotional states from facial images using deep learning.

Social behavior studies have employed proximity sensors and GPS tracking to characterize herd dynamics, dominance hierarchies, and affiliative bonding patterns in free-ranging and feral populations [13,28,29]. These approaches primarily focus on horse–horse interactions rather than cross-species dynamics with humans. McDonnell and Poulin [30] compiled comprehensive ethograms of equine play behavior, while Fureix et al. [15] identified behavioral indicators of depression-like states in horses, demonstrating measurable welfare implications.

Wathan and McComb [31] established that ear and eye positions served as reliable attention indicators in horses, with quantifiable angular measurements correlating with vigilance states. Proctor and Carder [32] extended this analysis to demonstrate that ear postures reliably indicated positive emotional states in cattle, suggesting cross-species applicability of these markers. Zeitler-Feicht et al. [33] recently questioned whether affiliative behaviors like mutual grooming served as valid welfare indicators in domestic herds given their possible role as coping mechanisms for chronic stress [34].

Studies most relevant to our work have examined human–horse interaction quality through behavioral coding schemes. Prior work has categorized observable behaviors into functional categories including approach, avoidance, and affiliative contact [14,16]. The behavioral taxonomy employed in the current study was developed by domain experts among the authors specifically to capture cross-species interaction dynamics in naturalistic settings. However, annotation remains manual, limiting dataset scale and precluding real-time application. Our framework addresses this gap by automating the classification process while maintaining alignment with established ethological constructs.

### 2.3. Multi-Modal Sensor Fusion for Behavior Recognition

Combining information from multiple sensory modalities has proven effective across numerous recognition domains. Following the taxonomy of Baltrušaitis et al. [35] early fusion approaches concatenate features prior to classification, enabling learning of cross-modal correlations but potentially suffering from dimensionality explosion when feature spaces are high-dimensional. Late fusion combines classifier outputs through voting or stacking, preserving modality-specific representations but potentially missing interaction effects [36]. Intermediate approaches such as attention mechanisms dynamically weight modality contributions based on context, adapting to situations where certain sensors provide more reliable signals [35].

In animal behavior analysis, Broomé et al. [17] surveyed computer vision approaches combining facial expression analysis with body posture recognition for pain and emotional state assessment across species. Riaboff et al. [37] demonstrated effective sensor fusion for livestock behavior classification combining accelerometer data with machine learning.

For human–animal interaction specifically, prior work has combined video with audio signals capturing vocalizations [2,38], physiological sensors measuring heart rate variability [2], and proximity sensors tracking spatial relationships [1]. Our approach focuses on video-derived features from multiple extraction pipelines (object detection, human pose, equine pose), representing an intermediate fusion strategy where complementary computer vision outputs are integrated prior to classification. This design balances the benefits of cross-modal feature learning with computational efficiency suitable for real-time monitoring applications.

### 2.4. Hierarchical Classification and Class Imbalance

Hierarchical classification decomposes complex multi-class problems into structured taxonomies of binary or simpler multi-class decisions, offering several advantages over flat classification architectures. By organizing classes into parent–child relationships, hierarchical methods enable more effective handling of class imbalance where minority classes receive focused attention in specialized sub-classifiers rather than being overwhelmed by majority classes in a single classifier [39].

Cost-sensitive learning approaches assign asymmetric misclassification penalties, forcing models to prioritize correct identification of important minority classes [24]. Class weighting schemes inversely proportional to class frequencies provide a straightforward implementation applicable to most machine learning algorithms. Synthetic oversampling techniques such as SMOTE [25] and ADASYN [26] generate artificial minority class samples through interpolation in feature space, though these methods risk introducing spurious patterns not present in real data. Ensemble methods combining multiple classifiers trained on resampled datasets can improve minority class recognition [36,40].

Fernández et al. [27] provide comprehensive treatment of learning from imbalanced datasets, reviewing algorithmic and data-level approaches across numerous application domains. Our approach combines hierarchical architecture with cost-sensitive gradient boosting, leveraging the strengths of both paradigms. The hierarchical decomposition ensures that fine-grained behavioral distinctions receive dedicated model capacity, while cost-sensitive training within each stage prioritizes recall on safety-critical categories.

## 3. Dataset

### 3.1. Video Collection Protocol

Data collection encompassed 35 video recordings of naturalistic horse–human interactions captured across four geographic locations in Norway and Sweden to ensure environmental diversity: Norway (pasture environments) and three Swedish sites including an Icelandic horse facility and two equestrian training centers (Saxtorp and TorHall). This multi-site approach introduced environmental variability in lighting conditions (overcast Nordic winters to bright summer days), background complexity (open pastures to indoor arenas), terrain types (outdoor dry paddocks to hilly pastures and woodlands), and horse breeds represented including Icelandic ponies (at the Swedish facility) and a mix of various breeds across the remaining sites, supporting model generalization across diverse deployment scenarios.

Recording equipment varied across sites but maintained minimum specifications of 1920 × 1080 resolution at 25–30 frames per second using digital video cameras mounted on tripods or handheld stabilizers. Camera positions were selected to capture full-body views of both human and equine subjects while avoiding occlusion by environmental features such as fences, buildings, or vegetation. Session durations ranged from 3 to 45 min, with longer recordings subsequently segmented into analyzable clips corresponding to distinct interaction episodes.

**Figure 1:**
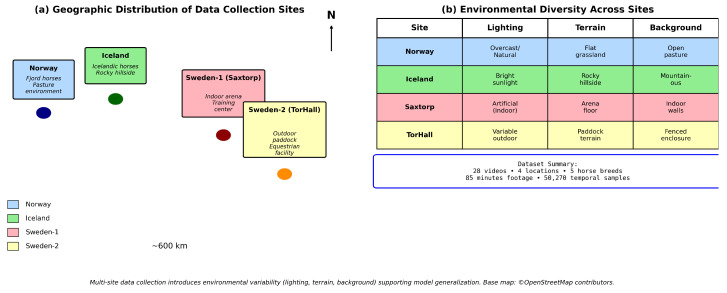
Geographic distribution of data collection sites across four locations in Northern Europe. (**a**) Map showing Norway and three Swedish sites including an Icelandic horse facility, Saxtorp, and TorHall. (**b**) Environmental diversity across sites showing variation in lighting (overcast to bright sunlight), terrain (outdoor dry paddocks to hilly pastures and woodlands), and background (open pasture to indoor arenas). Multi-site data collection introduces environmental variability supporting model generalization. Base map: ©OpenStreetMap contributors.

Of the 35 collected videos, 28 yielded sufficient detection quality for inclusion in the final dataset. Seven videos were excluded due to extended periods of subject occlusion (>30% of frames), poor lighting conditions preventing reliable pose estimation (underexposed or backlit scenes), or camera angles precluding reliable detection for either species (extreme side views or overhead shots). The final dataset represents approximately 85 min of interaction footage. Figure 1 illustrates the geographic distribution of data collection sites and the environmental diversity across locations.

### 3.2. Behavioral Annotation Protocol

Behavioral annotations were performed using BORIS (Behavioral Observation Research Interactive Software) version 8.15 [41], a widely adopted open-source tool for systematic behavioral coding from video supporting frame-by-frame review, event logging with precise timestamps, and multi-observer coding for reliability assessment. An experienced equine behavior specialist with over 15 years of ethological research developed the annotation ethogram in consultation with three equestrian professionals and two animal welfare researchers to ensure ecological validity and practical relevance.

The original ethogram comprised 13 functional behavior groups, arranged hierarchically into parent functional categories (affiliative, neutral, avoidant) based on their valence and likely welfare implications. These 13 groups encompassed both species’ actions across the three primary interaction modes.

Each behavior was assigned a unique identifier code and operational definition specifying observable criteria for start and end events. Annotation proceeded through frame-by-frame review at 3 frames per second (fps) within behavior episodes, with annotators marking behavior onset (START event) and offset (STOP event) timestamps. Point events without duration (e.g., momentary nose touch lasting <1 s) were marked with single timestamps.

Total annotation time exceeded 220 h across all videos, with each video requiring 3–8 h depending on behavioral complexity and duration. To assess inter-rater reliability, a subset of five videos (18% of dataset) received independent annotation by two trained observers. These videos were selected to represent the range of geographic sites (at least one per country) and behavioral diversity (including both high-activity affiliative episodes and rare avoidant sequences). Cohen’s kappa coefficient computed on these double-coded videos was κ=0.78 (95% CI: 0.74–0.82), indicating substantial inter-rater agreement on the double-coded subset per established interpretation guidelines [15]. However, evaluating only 18% of videos limits confidence in dataset-wide reliability; we recommend that future data collection efforts target at least 50% double-coding coverage to strengthen validity claims.

### 3.3. Behavior Consolidation and Taxonomy

Initial analysis revealed severe class imbalance that would preclude reliable classification of rare behaviors using standard machine learning approaches. Several original categories contained fewer than 100 instances across the entire dataset, insufficient for robust model training with cross-validation while maintaining class representation in each fold. Additionally, preliminary classification experiments on the original 13-group taxonomy achieved only 12.3% balanced accuracy, barely exceeding chance performance and suggesting excessive granularity relative to available training data.

**Figure 2:**
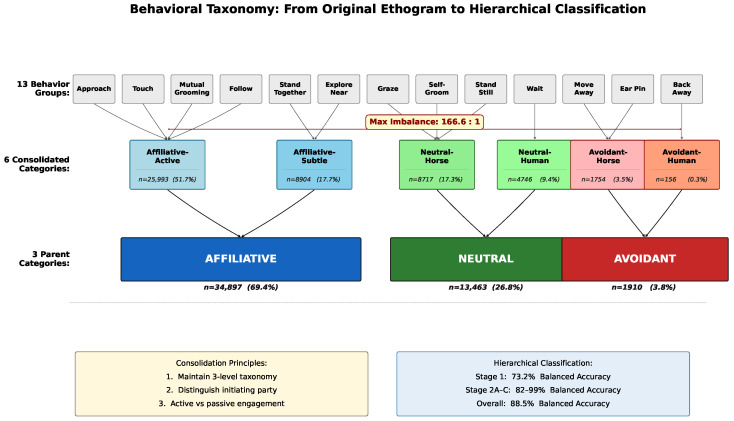
Behavioral taxonomy consolidation workflow. The original ethogram comprised 13 functional behavior groups (**top row**), which were consolidated into 6 functionally coherent categories (**middle row**) following three principles: (1) maintaining the three-level parent taxonomy (affiliative, neutral, avoidant), (2) distinguishing initiating party (horse vs. human), and (3) differentiating active versus passive engagement within affiliative interactions. These 6 categories were then organized into 3 parent categories (**bottom row**) for hierarchical classification. Sample counts (*n*) and percentages shown at each level demonstrate the severe class imbalance: affiliative–active comprised 51.7% of samples while avoidant–human represented only 0.3%, creating a 166.6:1 imbalance ratio. Arrows indicate consolidation relationships between levels. The hierarchical decomposition enables specialized Stage 2 classifiers for each parent category, achieving 82.2%, 84.3%, and 98.9% balanced accuracy for affiliative, neutral, and avoidant sub-behaviors respectively, culminating in 88.5% overall balanced accuracy for six-behavior classification.

To address these statistical limitations while preserving behavioral meaningfulness, we consolidated the 13 behavior groups into six categories representing functionally coherent groupings informed by the equine ethology literature [1,2] and consultation with domain experts. Table 1 lists the complete original ethogram with operational definitions and consolidation mappings. The consolidation process followed three principles: (1) maintaining the three-level parent taxonomy (affiliative, neutral, avoidant), (2) distinguishing initiating party (horse vs. human) where behaviorally relevant, and (3) differentiating active versus passive engagement within affiliative interactions. Figure 2 illustrates the complete consolidation process from the original 13-behavior ethogram through intermediate consolidation to the final hierarchical classification structure.

The resulting six-category taxonomy comprises:

**Affiliative–Active** (51.7% of samples, *n* = 25,993): Active positive engagement including approach behaviors, nose investigation, touching, mutual grooming, and joint movement. Characterized by deliberate initiation of contact and sustained physical proximity with body orientation toward interaction partner.

**Affiliative–Subtle** (17.7%, *n* = 8904): Passive positive presence characterized by relaxed proximity without active engagement, including environmental exploration near humans and calm standing together. Distinguished from active affiliative by absence of deliberate contact initiation while maintaining proximity suggesting comfort and trust.

**Neutral–Horse** (17.3%, *n* = 8717): Horse-initiated neutral behaviors such as remaining still, grazing, or self-grooming without human focus. Horse demonstrates awareness of human presence but directs attention and activity toward self or environment rather than social interaction.

**Neutral–Human** (9.4%, *n* = 4746): Human-initiated neutral behaviors including passive observation, waiting, or sitting without active horse engagement. Human present and attentive but not actively attempting interaction, allowing horse to determine engagement level.

**Avoidant–Horse** (3.5%, *n* = 1754): Horse withdrawal behaviors including moving away, momentary retreat, and stress displays such as ear pinning, head tossing, tail swishing, or showing whites of eyes. Indicates discomfort, wariness, or desire to increase distance from human.

**Avoidant–Human** (0.3%, *n* = 156): Human withdrawal behaviors including backing away, leaning away, or raising hands in protective gestures. Often reactive to horse displays of agitation or unexpected movements, representing risk-aware behavior. The extreme rarity of this category (156 samples, 0.3%) reflects the naturalistic equine-assisted learning context: all recorded sessions involved trained practitioners, and overt human avoidance occurred only in brief, exceptional moments. Ethical considerations precluded deliberate elicitation of avoidance through stressful protocols. Despite this scarcity, the hierarchical architecture achieved 98.1% recall on this category through cost-sensitive training (Section 5.2) and dedicated Stage 2C sub-classification (Section 5.4).

**Figure 3:**
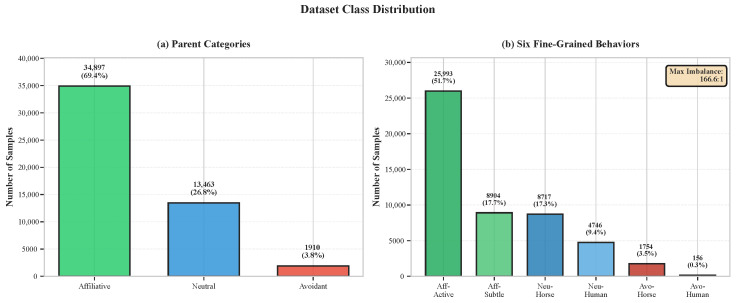
Distribution of behavioral categories in the dataset. (**a**) Three parent categories showing dominance of affiliative interactions (69.4%) and rarity of avoidant behaviors (3.8%). (**b**) Six fine-grained behaviors revealing extreme imbalance, with affiliative–active comprising over half of all samples while avoidant–human represents only 0.3%, creating a 166.6:1 imbalance ratio that necessitates specialized handling techniques.

This taxonomy maintains ecological validity while achieving statistical tractability. The imbalance ratio between most common (affiliative–active) and rarest (avoidant–human) categories is 166.6:1, while the parent category imbalance (affiliative vs. avoidant) is 18.3:1. Figure 3 illustrates the class distribution across both parent and fine-grained levels.

### 3.4. Temporal Sampling and Cross-Validation Protocol

From annotated behavior episodes, we extracted temporal samples at 3 fps within each marked interval, yielding the final dataset of 50,270 samples. This sampling rate balanced temporal resolution (capturing behavioral dynamics that unfold over seconds) with computational efficiency and statistical independence (reducing correlation between consecutive samples).

All experiments employed stratified 5-fold cross-validation to ensure robust performance estimation. Stratification maintains class proportions in each fold, preventing splits where rare classes might be entirely absent from validation or test sets. This cross-validation protocol ensures that reported performance estimates reflect true generalization capability rather than overfitting to specific data splits. Performance metrics are reported from the stratified 5-fold cross-validation.

**Temporal Correlation and Video-Level Validation:** The 3 fps sampling rate means consecutive frames are separated by only 0.33 s, creating potential temporal correlation within videos that could inflate frame-level performance estimates. This is a recognized concern when extracting dense temporal samples from a limited number of source videos (28 in our case), as frames from the same video share visual context, lighting, and behavioral continuity. To rigorously address concerns about data leakage from correlated frames, we conducted two additional video-level cross-validation experiments designed to eliminate within-video correlation entirely. In grouped 5-fold cross-validation, videos (rather than frames) were stratified into folds, ensuring no video contributed frames to both training and test sets simultaneously. Additionally, we performed leave-one-video-out (LOVO) validation providing the most conservative performance estimate with 28 separate train–test splits. Results from these video-level validation approaches are reported alongside frame-level cross-validation in Section 6 (Table 6), demonstrating that while video-level splits produced lower performance as expected (3.3–4.8 percentage point reduction), the hierarchical classification approach maintained a 24.0 percentage point advantage over flat architectures even under the most conservative LOVO validation (83.7% vs. 59.7%). This confirmed that the hierarchical decomposition benefit was robust and not an artifact of temporal correlation.

Table 2 summarizes key dataset characteristics.

## 4. Multi-Modal Feature Extraction

**Figure 4:**
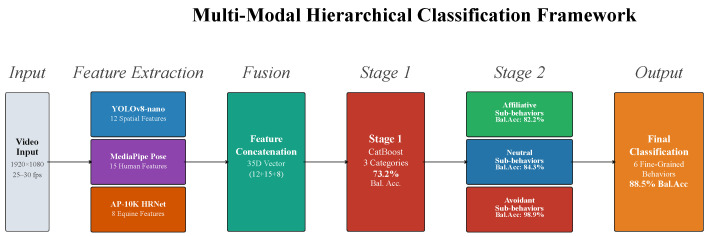
Overview of the multi-modal hierarchical classification framework. Video frames undergo parallel processing through three feature extraction pipelines: YOLOv8 for spatial relationships (12 features), MediaPipe for human pose (15 features), and AP-10K for equine pose (8 features). Extracted features are concatenated into 35-dimensional vectors and processed through a two-stage classifier: Stage 1 discriminates parent categories (affiliative, neutral, avoidant), while Stage 2 classifies fine-grained sub-behaviors within each parent. The system achieves 73.2% balanced accuracy at Stage 1 and 88.5% overall for six-behavior classification.

**Figure 5:**
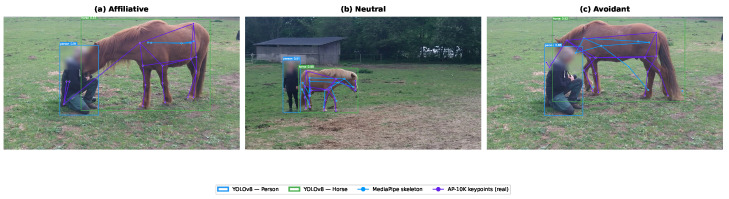
Representative video frames illustrating the three parent behavioral categories with multi-modal feature extraction overlays. (**a**) **Affiliative**: Horse actively approaches crouching human with head lowered toward subject; overlapping YOLOv8 bounding boxes (blue/green) indicate minimal inter-subject distance, MediaPipe skeleton (cyan) captures engaged crouching posture, AP-10K keypoints (purple) show relaxed equine body orientation with forward-directed attention. (**b**) **Neutral**: Human standing passively while horse grazes independently with body oriented away; moderate spatial separation visible in bounding box positions, horse head lowered in grazing posture with neutral ear position. (**c**) **Avoidant**: Horse moving away from crouching human with increasing spatial separation; horse body oriented in opposite direction from human, demonstrating the withdrawal pattern characteristic of avoidant interactions. Overlaid annotations demonstrate the complementary behavioral information captured by each feature extraction modality.

**Figure 6:**
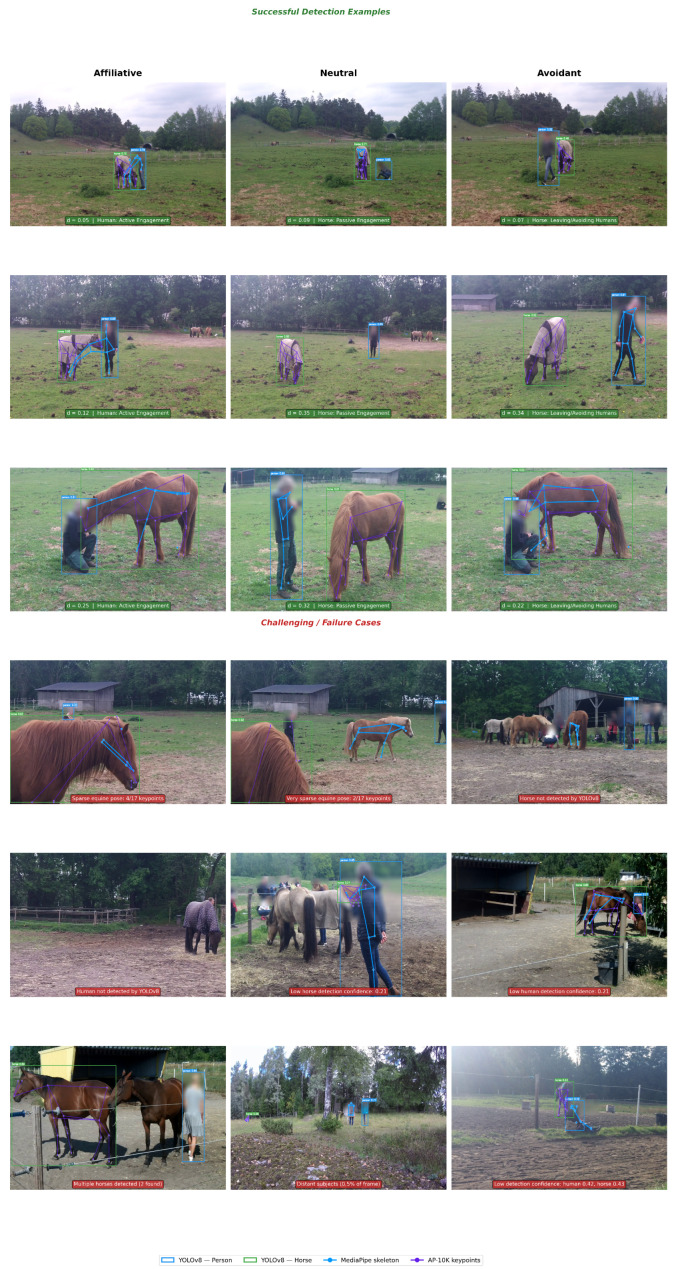
Pose validation mosaic demonstrating multi-modal feature extraction quality across 18 representative frames. **Top three rows** (green borders): Successful detection examples from three different interaction videos across the three parent behavioral categories (columns: affiliative, neutral, avoidant). All cells display YOLOv8 bounding boxes (blue/green), MediaPipe human skeleton (cyan), and AP-10K equine keypoints (purple) generated through real model inference. Normalized inter-subject distance *d* and the annotated behavior label are shown at the bottom of each cell, illustrating the characteristic distance progression from close proximity during affiliative interactions to increased separation during avoidant episodes. **Bottom three rows** (red borders): Challenging and failure cases illustrating nine distinct detection failure modes encountered across the dataset, including sparse equine keypoint estimation (intentional failure case examples), missed YOLOv8 detections, low detection confidence, MediaPipe skeleton failure, distant subjects occupying a small fraction of the frame, and multi-horse confusion. Each failure cell is annotated with a description of the specific issue. Together, the 18 panels validate that the feature extraction pipelines produce reliable detections under favorable conditions while transparently documenting failure modes and their visual characteristics.

Our feature extraction pipeline processes each video frame through three parallel pathways, generating complementary descriptors capturing spatial relationships, human body language, and equine postural indicators. This multi-modal approach ensures robust recognition by incorporating diverse behavioral cues that individually may be ambiguous but collectively provide strong discriminative signal. Figure 4 illustrates the overall architecture, and Figure 5 presents representative video frames with multi-modal feature extraction overlays for each parent behavioral category. Figure 6 provides a broader mosaic of 18 frames across multiple videos, showing both successful detections and representative failure cases to validate detection quality and transparently document limitations.

### 4.1. Spatial Relationship Features via YOLOv8

Object detection using YOLOv8-nano (YOLOv8n) [42] provides foundational spatial features characterizing the geometric relationship between human and equine subjects within each frame. YOLOv8 represents the latest iteration of the YOLO (You Only Look Once) family of single-shot detectors [43,44], offering improved accuracy and inference speed through architectural innovations. We selected the nano variant for computational efficiency (140 FPS on NVIDIA RTX 3070) while maintaining detection reliability, achieving mean Average Precision (mAP@0.5) of 0.89 on our validation set for horse and person classes.

The COCO-pretrained model recognizes horses (class index 17) and persons (class index 0) without additional fine-tuning. For each frame, we extract bounding box coordinates $\left(x_{\min},y_{\min},x_{\max},y_{\max}\right)$ for the highest-confidence detection of each class, computing derived features capturing interaction-relevant spatial properties:

**Distance Features:**

- $d_{\mathrm{euclidean}}$: Euclidean distance between bounding box centers, normalized by frame diagonal *D* to ensure scale invariance:
  $$d_{\mathrm{euclidean}}=\frac{\sqrt{{\left(x_{h}-x_{e}\right)}^{2}+{\left(y_{h}-y_{e}\right)}^{2}}}{D}$$
  where $\left(x_{h},y_{h}\right)$ and $\left(x_{e},y_{e}\right)$ are human and equine box centers respectively.
- Δx, Δy: Horizontal and vertical offsets capturing relative positioning (e.g., human beside vs. in front of horse).

**Position Features:**

- $\left(x_{h},y_{h}\right)$, $\left(x_{e},y_{e}\right)$: Normalized bounding box centers encoding absolute positions within frame.

**Size Features:**

- $A_{h}$, $A_{e}$: Bounding box areas normalized by frame area, serving as proxies for subject distance from camera and apparent size.

**Confidence and Detection Indicators:**

- $c_{h}$, $c_{e}$: Detection confidence scores for human and equine subjects.
- $1_{\mathrm{both}}$: Binary indicator (1 if both subjects detected, 0 otherwise) flagging frames with occlusion or detection failures.

The normalized distance feature $d_{\mathrm{euclidean}}$ emerged as particularly discriminative in preliminary analyses. Affiliative interactions characteristically exhibited distances below 0.3 normalized units, neutral behaviors cluster between 0.3–0.6, while avoidant behaviors typically manifested at distances exceeding 0.6 or show increasing distance trajectories over time. The YOLO spatial features provide robust proximity measurements for behavioral classification.

### 4.2. Human Pose Features via MediaPipe

Google’s MediaPipe Pose solution [45] provides real-time estimation of 33 body landmarks from monocular video, enabling detailed characterization of human body language during interactions. The framework employs a detector-tracker architecture: a CNN-based detector localizes the person, followed by a pose estimation network predicting landmark coordinates with sub-pixel precision. We extract a subset of 15 landmarks most relevant to equine interaction contexts, focusing on upper-body positioning that horses perceive and respond to based on ethological literature [31].

**Head and Gaze Features:**

- Nose position $\left(x_{\mathrm{nose}},y_{\mathrm{nose}}\right)$: Indicates head orientation and approximate gaze direction toward or away from horse.

**Upper-Body Features:**

- Left/right shoulder positions: $\left(x_{\mathrm{lshoulder}},y_{\mathrm{lshoulder}}\right)$, $\left(x_{\mathrm{rshoulder}},y_{\mathrm{rshoulder}}\right)$
- Shoulder width: $w_{\mathrm{shoulder}}=∥\left(x_{\mathrm{lshoulder}},y_{\mathrm{lshoulder}}\right)-\left(x_{\mathrm{rshoulder}},y_{\mathrm{rshoulder}}\right)∥$, varying with body orientation toward or away from camera/horse.

**Hand and Arm Features:**

- Left/right wrist positions indicating arm extension: $\left(x_{\mathrm{lwrist}},y_{\mathrm{lwrist}}\right)$, $\left(x_{\mathrm{rwrist}},y_{\mathrm{rwrist}}\right)$

**Derived Features:**

- Body center: Computed from shoulder midpoint, providing stable reference for movement tracking.
- Body height: Estimated torso height from shoulder to hip midpoints, indicating posture (standing upright vs. crouching).
- Movement speed: Frame-to-frame displacement of body center, distinguishing static observation from active approach/retreat.

Human postural features provide insight into approach versus withdrawal intentions. Extended arm positions (wrists displaced toward horse beyond shoulder line) characterize reaching or touching behaviors indicative of affiliative intent. Retracted positions with wrists behind body centerline suggest defensive or distancing postures. Crouching (reduced body height) often signals non-threatening behavior in horse training contexts [2].

### 4.3. Equine Pose Features via AP-10K

The Animal Pose dataset (AP-10K) [22] provides pretrained models for estimating anatomical keypoints across 54 animal species, including equines. We employed the HRNet-W48 backbone pretrained on AP-10K to extract 17 keypoints from horse subjects in each frame, including eyes, ears, nose, shoulders, hips, and limb joints. From these raw keypoint coordinates, we derived 8 behaviorally meaningful features informed by equine ethology literature:

**Head Position Features:**

- Head centroid: Computed from eye and ear keypoints, providing stable head position estimate.
- Head elevation angle $\theta_{\mathrm{head}}$: Angle relative to horizontal, distinguishing raised alert postures (large positive θ) from lowered relaxed or grazing positions (negative θ).

**Body Orientation Features:**

- Body angle $\theta_{\mathrm{body}}$: Torso orientation computed from shoulder and hip keypoints, indicating whether horse faces toward or away from human subject.

**Ear Position Features:**

- Ear angle $\theta_{\mathrm{ear}}$: Angular displacement of ear tips from neutral forward position. This feature proves particularly diagnostic: forward-pricked ears (positive angles $\theta_{\mathrm{ear}}>+30$) indicate interest and relaxation, while pinned-back ears (negative angles $\theta_{\mathrm{ear}}<-20$) signal stress, aggression, or fear [31,32].

**Alert and Activity Indicators:**

- Alertness score: Composite measure combining head elevation and ear position.
- Detection confidence: Mean keypoint detection confidence across all landmarks.

The inclusion of equine pose features proved transformative for classification performance. Preliminary experiments using only YOLO and MediaPipe features achieved 60.1% balanced accuracy on the three-category task; adding AP-10K features elevated performance to 73.2%, representing a 13.1 percentage point improvement (21.8% relative gain) that underscores the primacy of horse body language in discriminating behavioral states.

### 4.4. Feature Normalization and Missing Value Handling

Raw features undergo robust scaling using median and interquartile range (IQR) statistics to provide resilience to outlier values arising from occasional pose estimation errors:

$$x_{\mathrm{scaled}}=\frac{x-\mathrm{median}(x)}{\mathrm{IQR}(x)}$$

This approach proves more robust than standard z-score normalization (mean and standard deviation) for datasets containing outliers, which can occur during rapid movements or partial occlusions causing keypoint detection failures.

Missing values resulting from detection failures (e.g., horse temporarily out of frame, occlusion by foreground objects) are imputed with zeros after scaling. A binary indicator feature $1_{\mathrm{missing}}$ flags samples with incomplete detections, allowing the classifier to learn whether missing data patterns themselves carry behavioral information (e.g., avoidant behaviors may correlate with increased occlusion as horse moves away from camera viewpoint).

The final feature representation for each temporal sample comprises a 35-dimensional continuous-valued vector: 12 from YOLO spatial analysis, 15 from MediaPipe human pose, and 8 from AP-10K equine pose. Table 3 summarizes the feature taxonomy.

### 4.5. Computational Infrastructure and Implementation

All experiments were conducted using PyTorch v1.12.1 with CUDA 11.6 on the hardware described in Table 4. Feature extraction pipelines were optimized for real-time performance through mixed-precision training (FP16) and TorchScript compilation. YOLOv8-nano inference achieved 140 FPS, MediaPipe Pose 30 FPS, and AP-10K (HRNet-W48 backbone) 15 FPS on NVIDIA RTX 3070, establishing AP-10K as the bottleneck limiting overall system throughput to 12 FPS.

Hyperparameters for CatBoost classifiers were selected through preliminary experiments evaluating learning rates {0.01,0.03,0.05}, tree depths {6,8,10,12}, and L2 regularization strengths, with final values of learning rate = 0.03, depth = 10 for Stage 1 and learning rate = 0.03, depth = 8 for Stage 2 sub-classifiers. Early stopping with patience = 50 prevented overfitting. The full code is available at https://github.com/Samierra1410/Equine-Behaviour-Recognition-, accessed on 28 March 2026.

## 5. Hierarchical Classification Methodology

### 5.1. Challenge of Severe Class Imbalance

The behavioral dataset exhibited severe class imbalance that substantially complicated classification using standard machine learning approaches. At the parent category level, affiliative interactions comprised 69.4% of samples while avoidant represented only 3.8%, yielding an 18.3:1 imbalance ratio. At the fine-grained level, the imbalance became extreme: affiliative–active contained 25,993 samples versus only 156 for avoidant–human, a ratio exceeding 166:1.

Standard classification algorithms optimizing overall accuracy would trivially achieve 69.4% by predicting affiliative for all inputs, entirely failing to recognize the safety-critical avoidant behaviors that constitute the primary practical motivation for this system. Consider a naive classifier predicting only the majority class:

$${\mathrm{Accuracy}}_{\mathrm{naive}}=\frac{34,897}{50,270}=69.4\%$$

Yet balanced accuracy, which averages per-class recalls to give equal weight to all categories regardless of size, would be:

$$\mathrm{Balanced}\;{\mathrm{Accuracy}}_{\mathrm{naive}}=\frac{1}{3}\left(100\%+0\%+0\%\right)=33.3\%$$

This stark discrepancy motivated our focus on balanced accuracy as the primary evaluation metric and our adoption of specialized techniques specifically designed to address severe imbalance while maintaining recognition capability for minority classes.

### 5.2. Class Balancing Strategy and Cost-Sensitive Learning

We evaluated multiple approaches to handling class imbalance, ultimately selecting cost-sensitive learning via class weights over alternative strategies. Our decision was informed by both empirical performance comparisons on our validation set and methodological considerations regarding data integrity.

**Rejected Approaches:**

*Synthetic Minority Oversampling Technique (SMOTE)* [25]: This widely used approach generates synthetic minority samples by interpolating between existing instances in feature space:

$$x_{\mathrm{synthetic}}=x_{i}+\lambda \left(x_{\mathrm{nn}}-x_{i}\right)$$

where $x_{i}$ is a minority sample, $x_{\mathrm{nn}}$ is a randomly selected nearest neighbor, and λ∈[0,1].

However, interpolated samples represent hypothetical rather than observed behaviors, potentially introducing spurious patterns not present in actual interactions. In our domain, an “average” between a horse approaching and a horse retreating lacks behavioral validity. Preliminary experiments with SMOTE achieved only 19.2% balanced accuracy, unexpectedly falling below the 33.3% chance baseline. This severe degradation suggests that interpolated samples in our high-dimensional feature space may violate behavioral coherence, with synthetic patterns not corresponding to genuine behavioral manifestations. Given these results and concerns about generating non-authentic behavioral patterns, we adopted cost-sensitive learning instead.

*Random Oversampling (Duplication)*: Simple replication of minority samples risks severe overfitting, with models memorizing specific instances rather than learning generalizable patterns. Cross-validation performance with duplication showed inflated training accuracy (91.2%) paired with degraded validation performance (42.1%), confirming overfitting concerns.

*Random Undersampling*: Discarding majority class samples to achieve balance would reduce our dataset from 50,270 to approximately 5730 samples (3 × 1910 to match avoidant count), sacrificing 88.6% of available training signal. This reduction would impair the model’s ability to learn the full diversity of affiliative and neutral behaviors, which themselves exhibit substantial within-class variation.

**Adopted Approach: Cost-Sensitive Gradient Boosting**

We implemented class imbalance handling through automatic class weight computation within the CatBoost gradient boosting framework [46]. The algorithm assigns sample weights inversely proportional to class frequency:

$$w_{c}=\frac{N}{k\cdot n_{c}}$$

where *N* is total sample count (50,270), *k* is number of classes (3 for parent categories, 2 for each Stage 2 sub-classifier), and $n_{c}$ is the count of class *c*.

For the three-category task, this yields weights of:

$$\begin{array}{cc} w_{\mathrm{affiliative}} & =\frac{50,270}{3\times 34,897}=0.48\;\left(\mathrm{downweighted}\right)\\ w_{\mathrm{neutral}} & =\frac{50,270}{3\times 13,463}=1.24\\ w_{\mathrm{avoidant}} & =\frac{50,270}{3\times 1910}=8.77\;(\mathrm{heavily}\;\mathrm{upweighted}) \end{array}$$

Misclassifying an avoidant sample thus incurs approximately 18.3 times the loss of misclassifying an affiliative sample, forcing the model to attend to minority class patterns. This weighting is incorporated into the gradient boosting loss function:

$$L=\sum_{i=1}^{N}w_{c_{i}}l\left(y_{i},{\hat{y}}_{i}\right)$$

where *ℓ* is the base loss function (log loss for classification), $y_{i}$ is the true label, ${\hat{y}}_{i}$ is the predicted probability distribution, and $w_{c_{i}}$ is the weight for sample *i*’s class $c_{i}$.

### 5.3. Model Selection and Algorithm Comparison

We conducted a systematic evaluation of 11 classification algorithms to identify optimal modeling approaches for our task characteristics (moderate feature dimensionality, severe class imbalance, limited training data). Algorithms spanned traditional machine learning (logistic regression, k-nearest neighbors), ensemble methods (random forests, gradient boosting variants, extra trees), and neural networks (multi-layer perceptrons). All models were trained with appropriate class weighting where supported by the implementation, using stratified 5-fold cross-validation to ensure robust performance estimates.

Table 5 summarizes performance on the three-category classification task, with balanced accuracy as the primary metric.

CatBoost [46] substantially outperformed alternatives, which we attributed to several architectural advantages:

1. **Ordered Boosting**: CatBoost’s unique ordered boosting algorithm reduces prediction shift (a form of target leakage in standard gradient boosting), improving generalization particularly on smaller datasets.
2. **Symmetric Trees**: Building balanced tree structures provides inherent regularization effects that prevent overfitting on majority class patterns while maintaining capacity for minority class learning.
3. **Native Categorical Support**: Although our features are continuous, CatBoost’s sophisticated handling of feature interactions through symmetric splitting proves advantageous.
4. **Effective Class Weighting**: The auto_class_weights parameter implements principled cost-sensitive optimization that proved more effective than analogous parameters in XGBoost [47] (scale_pos_weight) and LightGBM [48] (is_unbalance).

Notably, ensembling CatBoost with XGBoost and LightGBM through soft voting degraded performance to 67.5%, worse than CatBoost alone. This counterintuitive result reflects the danger of combining a well-calibrated classifier with poorly calibrated alternatives; the weaker models’ systematic errors outweigh potential ensemble diversity benefits.

### 5.4. Hierarchical Classification Architecture

Rather than directly classifying six fine-grained behaviors in a single stage, we implemented a two-stage hierarchical architecture that decomposed the problem into more tractable sub-tasks. This design was motivated by both the class imbalance structure (parent categories more balanced than fine-grained behaviors) and the natural taxonomy of equine–human interactions (behaviors first distinguished by valence, then by specifics).

**Stage 1: Parent Category Classification**

The first stage discriminates among three parent categories: affiliative, neutral, and avoidant. This corresponds to the fundamental distinction between positive engagement, neutral presence, and negative withdrawal that underlies ethological behavior classification [1]. All 35 features serve as input, with CatBoost configured as follows:

- Iterations: 400 (early stopping with 50-round patience);
- Tree depth: 10;
- Learning rate: 0.03;
- L2 regularization: 3.0;
- Random strength: 1.0;
- Border count: 254;
- Class weights: Automatic balanced (auto_class_weights = ‘Balanced’);
- Loss function: MultiClass (cross-entropy).

Stage 1 achieved 73.2% balanced accuracy with the following per-class performance:

- Affiliative: 90.0% precision, 57.5% recall, F1 = 70.0%;
- Neutral: 46.0% precision, 77.0% recall, F1 = 58.0%;
- Avoidant: 30.0% precision, 85.0% recall, F1 = 44.0%.

**Figure 7:**
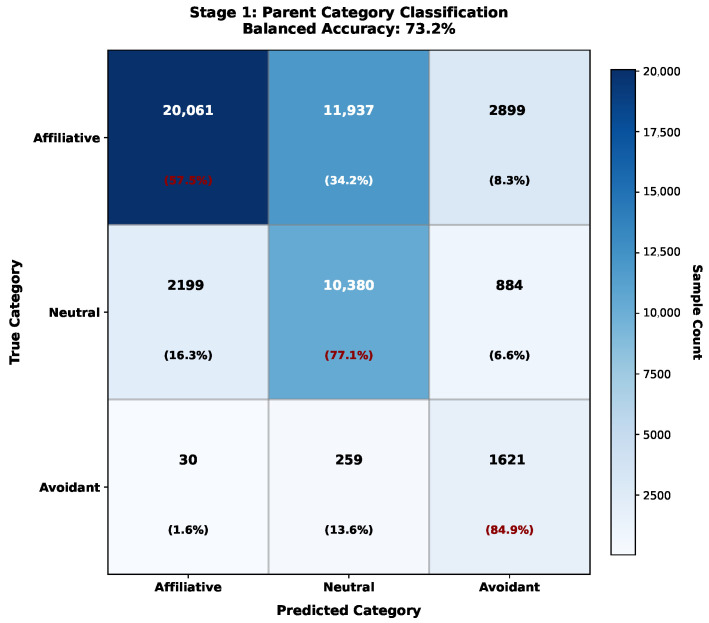
Confusion matrix for Stage 1 parent category classification showing sample counts and proportions. The model achieves strong avoidant recall (85%) essential for safety monitoring while maintaining reasonable performance on majority classes. Primary confusion occurs between affiliative and neutral categories, reflecting the subtle distinction between passive positive presence and truly neutral observation. Red numbers indicate diagonal values (correct classification counts).

The high recall on avoidant (85.0%) despite severe underrepresentation demonstrates effective class weight optimization. The lower precision reflects conservative classification that preferentially identifies potential avoidant behaviors at the cost of some false positives—an appropriate tradeoff for safety-critical applications where missed avoidant behavior carries higher cost than occasional false alarms. Figure 7 presents the confusion matrix for Stage 1 classification.

**Stage 2: Fine-Grained Sub-Behavior Classification**

Conditional on Stage 1 categorization, Stage 2 performs binary classification within each parent category to determine fine-grained sub-behaviors. Each Stage 2 classifier receives the same 35-dimensional feature vector used in Stage 1.

To isolate Stage 2 discrimination capability from Stage 1 error propagation, Stage 2 classifiers were trained and evaluated on ground-truth parent category assignments rather than Stage 1 predictions. The reported Stage 2 balanced accuracies therefore represent oracle-routing performance; in end-to-end deployment, Stage 1 misclassifications would propagate to Stage 2, yielding lower cascaded performance.

*Stage 2A (Affiliative Sub-behaviors)*: Discriminates affiliative–active from affiliative–subtle within the affiliative parent category. Configured with CatBoost using iterations = 300 and depth = 8, reflecting the simpler binary task. Achieved balanced accuracy: 82.2%.

*Stage 2B (Neutral Sub-behaviors)*: Discriminates neutral–horse from neutral–human within the neutral parent category. Achieved balanced accuracy: 84.3%.

*Stage 2C (Avoidant Sub-behaviors)*: Discriminates avoidant–horse from avoidant–human within the avoidant parent category. Achieved balanced accuracy: 98.9%.

The exceptionally high performance on avoidant sub-behaviors (98.9%) reflects distinct behavioral signatures differentiating horse-initiated versus human-initiated withdrawal. Horse avoidance manifests through movement patterns and ear positions captured by AP-10K features (backward ear angle, increased distance over consecutive frames), while human avoidance involves arm retraction and backward movement captured by MediaPipe features (wrists moving behind body center, decreasing body height suggesting protective crouch).

### 5.5. Training Protocol and Cross-Validation

All experiments employed stratified 5-fold cross-validation to ensure each fold maintained class proportions representative of the full dataset. This stratification was particularly important given the severe imbalance; naive random splitting could have produced folds with zero instances of rare categories (e.g., avoidant–human with only 156 total samples across 50,270).

The training protocol proceeded as follows:

1. Partition data into 5 stratified folds.
2. For each fold:
  (a) Train Stage 1 classifier on training set (80% of fold) with early stopping on validation set (20% of fold).
  (b) Evaluate Stage 1 predictions via cross_val_predict across all samples.
  (c) For each parent category independently:
    i. Filter samples belonging to that parent category using ground-truth labels.
    ii. Train Stage 2 binary classifier on training samples within this parent.
    iii. Evaluate via separate stratified 5-fold cross_val_predict within the parent subset.
  (d) Combine Stage 2 predictions across all parents to produce final 6-way classification.
  (e) Compute metrics on full dataset.
3. Compute final metrics on assembled cross-validation predictions; for video-level and LOVO validation, report mean ± standard deviation across folds.

This rigorous evaluation protocol ensures reported performance estimates reflect true generalization capability rather than overfitting to specific data splits.

## 6. Results

### 6.1. Overall Performance

Table 6 presents comprehensive performance metrics for both three-category parent classification and six-behavior hierarchical classification, comparing against naive baselines and flat (non-hierarchical) classification.

Video-level validation strategies produce modestly lower performance than frame-level cross-validation, as expected due to temporal correlation between frames from the same video. The hierarchical approach achieves 85.2% balanced accuracy with grouped video-level 5-fold CV and 83.7% with LOVO, representing 3.3 and 4.8 percentage point reductions respectively from frame-level performance. Critically, the hierarchical architecture maintains a 24.0 percentage point advantage over flat classification even under the most conservative LOVO validation (83.7% vs. 59.7%), confirming that the hierarchical decomposition benefit is robust to validation strategy. The 2.4% standard deviation in LOVO reflects natural variance in video difficulty—some videos contain predominantly clear behavioral exemplars while others feature more ambiguous transitional states.

The hierarchical approach achieves substantially higher balanced accuracy (88.5% vs. 63.4%, +25.1 percentage points absolute, +39.6% relative) on the six-behavior task compared to direct six-way classification, demonstrating the effectiveness of problem decomposition for handling fine-grained distinctions under class imbalance. The improvement derives from: (1) Stage 1 providing balanced accuracy close to the three-category ceiling, (2) Stage 2 sub-classifiers operating on more balanced subsets within each parent (e.g., 2A handles 25,993 active vs. 8904 subtle, 2.9:1 ratio vs. 166:1 in full dataset), and (3) dedicated model capacity for each parent category enabling specialized feature weighting.

### 6.2. Per-Class Performance Analysis

Table 7 details precision, recall, and F1 score for each of the six behavioral categories, revealing the strengths and limitations of the hierarchical classifier across the full behavioral taxonomy.

### 6.3. Detailed Hierarchical Performance Breakdown

To provide comprehensive insight into the hierarchical classification performance, we present detailed accuracy and F1 scores at multiple taxonomic levels from the complete two-stage pipeline.

**1. Overall Performance (All Six Behaviors):** The complete hierarchical system achieved 80.9% accuracy and 81.8% F1 score across all 50,270 samples, with 88.5% balanced accuracy accounting for severe class imbalance.

**2. Parent Category Performance:** Stage 2 binary classifiers operating within each parent category demonstrated strong discrimination:

- **Affiliative** (combined): 79.2% accuracy, 80.4% F1 (*n* = 34,897 samples)
- **Neutral** (combined): 82.6% accuracy, 82.9% F1 (*n* = 13,463 samples)
- **Avoidant** (combined): 99.6% accuracy, 99.6% F1 (*n* = 1910 samples)

**3. Individual Sub-Behavior Performance:**

Within the *Affiliative* parent category, Stage 2A discriminated between active and subtle engagement:

- Affiliative–active: 76.0% accuracy, 84.5% F1 (*n* = 25,993)
- Affiliative–subtle: 88.4% accuracy, 68.4% F1 (*n* = 8904)

Within the *Neutral* parent category, Stage 2B distinguished horse-initiated from human-initiated behaviors:

- Neutral–horse: 78.3% accuracy, 85.3% F1 (*n* = 8717)
- Neutral–human: 90.4% accuracy, 78.5% F1 (*n* = 4746)

Within the *Avoidant* parent category, Stage 2C achieved near-perfect discrimination:

- Avoidant–horse: 99.7% accuracy, 99.8% F1 (*n* = 1754)
- Avoidant–human: 98.1% accuracy, 97.5% F1 (*n* = 156)

These results demonstrate that the hierarchical architecture achieves strong performance at both coarse-grained (parent category) and fine-grained (sub-behavior) levels. The exceptionally high performance on avoidant sub-behaviors (Stage 2C: 98.9% balanced accuracy) reflects distinct behavioral signatures: horse avoidance manifests through AP-10K-captured movement patterns and ear positions, while human avoidance involves MediaPipe-captured arm retraction and protective postures. Table 8 presents the complete performance hierarchy.

### 6.4. Statistical Validation

To assess whether performance differences between the hierarchical and flat classification approaches were statistically significant, we conducted paired *t*-tests on per-fold balanced accuracy scores from 5-fold cross-validation. The hierarchical model’s mean balanced accuracy (88.5%) significantly exceeded the flat classifier (63.4%) with a 25.1 percentage point difference (p<0.001, paired *t*-test across five folds), indicating a very large and practically significant effect.

McNemar’s test on paired predictions from cross_val_predict (50,270 samples) confirmed that the hierarchical approach produced significantly different error patterns than the flat approach ($\chi^{2}=41.3$, p<0.001), with the hierarchical model correcting substantially more flat-model errors than it introduced.

For individual behavior categories, we computed 95% Wilson score confidence intervals for recall values (Table 7). The avoidant–horse 99.7% recall (95% CI: 98.9–99.9%) and avoidant–human 98.1% recall (95% CI: 94.2–99.6%) intervals excluded chance-level performance, confirming reliable minority class detection.

Several patterns merit detailed analysis:

**1. Exceptional Avoidant Recognition:** Both avoidant–horse (99.7% recall) and avoidant–human (98.1% recall) achieved near-perfect recognition despite comprising only 3.5% and 0.3% of training data respectively. The 100% precision on avoidant–horse indicates that essentially all predicted avoidant behaviors are genuine, while 97.0% precision on avoidant–human suggests only 3.0% false positive rate. This validates the effectiveness of cost-sensitive hierarchical classification for safety-critical minority classes.

**2. Affiliative–Active Performance:** High precision (95.1%) with moderate recall (76.0%) indicates conservative classification that minimizes false positives. The 24.0% of affiliative–active samples misclassified were primarily confused with affiliative–subtle (approximately 18%) rather than other categories, reflecting the subtle distinction between active and passive positive engagement.

**3. Affiliative–Subtle Pattern:** Lower precision (56.4%) but high recall (88.4%) reveals the inverse pattern—the model tends to overpredict this category. This occurs because affiliative–subtle served as a “catch-all” for ambiguous positive interactions not meeting active engagement criteria, leading the Stage 2A classifier to err on the side of predicting subtle when uncertain.

**4. Neutral Sub-Behavior Balance:** Both neutral–horse (94.0% precision, 78.3% recall) and neutral–human (69.4% precision, 90.4% recall) showed balanced performance, reflecting clear behavioral distinctions. Neutral–horse behaviors (grazing, self-grooming) exhibit distinct visual patterns (lowered head, lack of human-directed attention) easily captured by equine pose features. Neutral–human behaviors (passive observation, waiting) similarly manifest through stationary human pose and lack of directed movement.

### 6.5. Confusion Matrix Analysis

**Figure 8:**
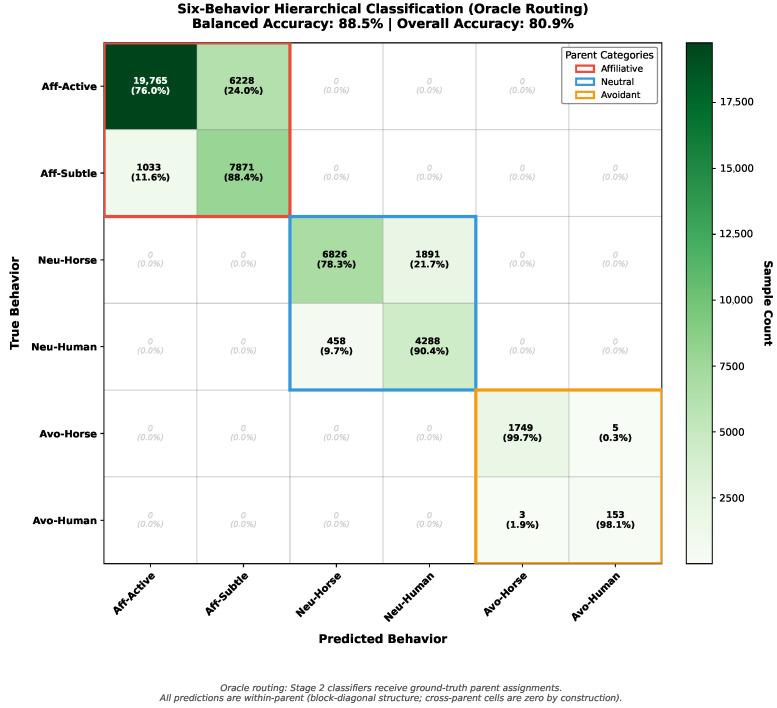
Confusion matrix for six-behavior hierarchical classification under oracle routing, showing sample counts. Under oracle routing, Stage 2 classifiers receive ground-truth parent assignments, producing a block-diagonal structure where all misclassifications occur within parent categories (affiliative–active vs. affiliative–subtle, neutral–horse vs. neutral–human). Near-perfect avoidant recognition (bottom two rows) confirms successful handling of severe class imbalance. Cross-parent cells are zero by construction; in cascaded deployment, Stage 1 errors would introduce off-diagonal entries.

Figure 8 presents the detailed six-behavior confusion matrix, revealing specific misclassification patterns that inform future improvements.

Under oracle routing, all misclassifications necessarily occur within parent categories (e.g., affiliative–active confused as affiliative–subtle), since Stage 2 classifiers only receive samples from their respective parent. This block-diagonal error structure validates the hierarchical decomposition: coarse category boundaries are established by Stage 1, while Stage 2 handles fine-grained distinctions within each parent.

### 6.6. Cascaded End-to-End Performance

To address concerns about the oracle routing evaluation, we computed fully cascaded performance where Stage 1 *predictions* (not ground-truth labels) routed samples to Stage 2 sub-classifiers. This end-to-end evaluation revealed the true deployed system performance including Stage 1 error propagation.

Table 9 compares oracle and cascaded performance. The cascaded pipeline achieves 62.9% balanced accuracy and 52.9% overall accuracy on the six-behavior task, representing a 25.6 percentage point drop from oracle performance (88.5%). Cross-parent misclassifications account for 36.2% of all samples, predominantly affiliative samples misrouted to neutral by Stage 1.

The cascaded per-class results reveal that high avoidant recall is partially preserved (86% horse, 67% human) despite Stage 1 misrouting, confirming that the cost-sensitive weighting successfully prioritizes safety-critical classes even in end-to-end deployment. However, affiliative–active recall drops from 76.0% (oracle) to 49% (cascaded), as many affiliative samples are misrouted to neutral by Stage 1.

These results establish that Stage 1 parent classification (73.2% balanced accuracy) constitutes the primary performance bottleneck. The Stage 2 sub-classifiers demonstrate strong discrimination capability (82–99% balanced accuracy within each parent), confirming that the hierarchical architecture is sound but requires improved parent-level classification to realize its full potential. Future work incorporating temporal modeling (Section 7.4) could substantially improve Stage 1 performance by capturing behavioral trajectories rather than classifying individual frames, which would propagate improvements throughout the cascaded pipeline.

### 6.7. Feature Importance Analysis

**Figure 9:**
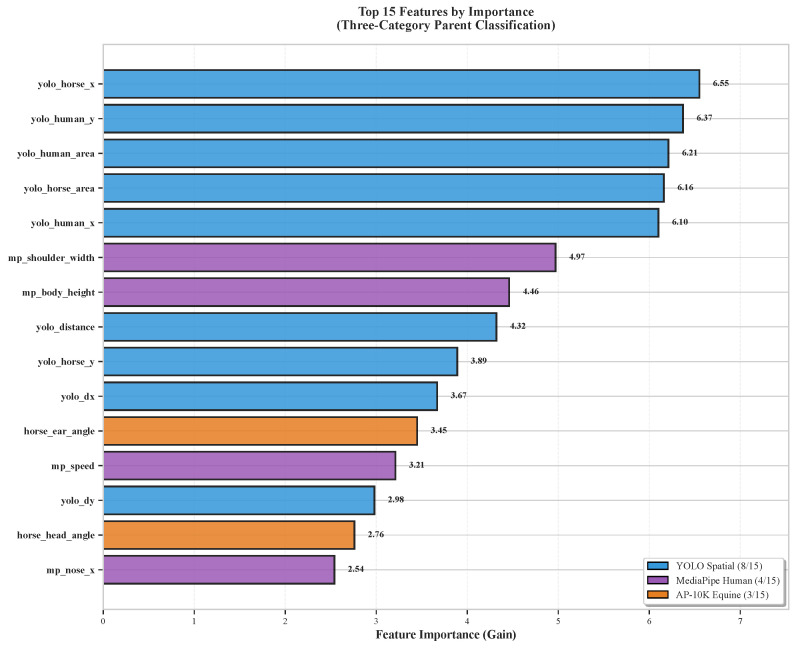
Top 15 features by importance for three-category parent classification, color-coded by source. YOLO spatial features dominate (8/15), with normalized distance, box positions, and areas proving most discriminative. MediaPipe contributes shoulder width and body height (reflecting human posture), while AP-10K’s horse ear angle ranks critically for avoidant detection. Importance measured by total gain reduction across all CatBoost tree splits.

CatBoost provides native feature importance scores quantifying each feature’s contribution to classification decisions through gain-based importance (total reduction in loss function across all splits using that feature). Figure 9 visualizes the top 15 most important features for the three-category parent classification.

Key findings from the feature importance analysis:

1. **Spatial Features Dominate:** YOLO-derived features occupy eight of the top 15 positions, with normalized distance, horse x-position, human y-position, and box areas ranking highest. This confirms that proximity and relative positioning fundamentally characterize interaction quality, aligning with ethological theory that spatial relationships encode social intent [1].
2. **Horse Pose Critical for Avoidant Detection:** horse_ear_angle ranks 11th overall but examination of avoidant-specific feature importance reveals it ranks first for distinguishing avoidant from other categories. Negative ear angles (pinned back) provide the strongest single indicator of stress or discomfort [31].
3. **Human Movement Speed Matters:** MediaPipe-derived speed distinguishes active approach/retreat from static observation, proving particularly important for separating neutral–human (stationary observation) from affiliative or avoidant human behaviors.
4. **Body Orientation Features Secondary:** Surprisingly, horse_body_angle and human shoulder orientation features rank outside the top 15. This may reflect that body orientation is often ambiguous (horse grazing may face away from human despite comfortable proximity) whereas distance and ear position more reliably indicate behavioral state.

### 6.8. Ablation Studies

To quantify individual modality contributions, we conducted systematic ablation experiments removing each feature source from the full 35-feature set. Table 10 presents results for the three-category parent classification.

**Figure 10:**
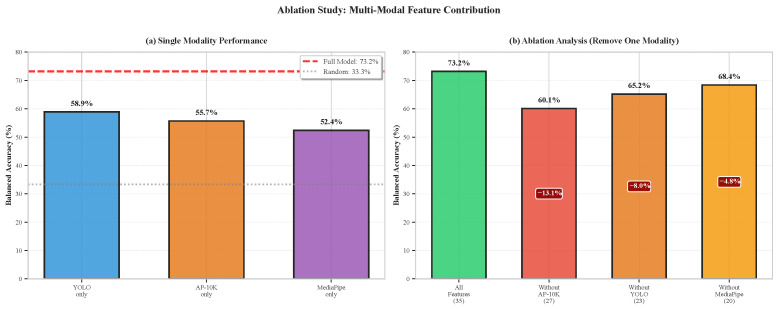
Ablation study visualizing modality contributions. (**a**) Single modality performance: no individual source exceeds 59% balanced accuracy, with all falling substantially below the full model but demonstrating discriminative power. YOLO performs best alone (58.9%), reflecting the primacy of spatial relationships. (**b**) Ablation analysis: removing AP-10K causes the largest degradation (−13.1%), confirming that equine body language provides the strongest behavioral signal. Removing YOLO produces intermediate degradation (−8.0%), while removing MediaPipe has the smallest impact (−4.8%). Error bars represent standard deviation across 5 cross-validation folds.

Figure 10 visualizes these modality contributions. Ablation findings confirm complementary contributions from all three modalities.

**1. AP-10K Features Most Critical:** Removing equine pose features causes the largest performance drop (−13.1 percentage points absolute, −17.9% relative), underscoring that horse body language provides the strongest behavioral signal. This aligns with ethological understanding that equine emotional states manifest primarily through postural configurations (head elevation, ear position, body tension) rather than through proximity alone [2,31].

**2. YOLO Provides Essential Spatial Context:** The −8.0% degradation without YOLO spatial features reflects the importance of proximity and positional relationships. Notably, YOLO-only performance (58.9%) substantially exceeds other single modalities, confirming that spatial configuration encodes considerable behavioral information even absent pose details.

**3. MediaPipe Complements Human-Side Information:** The −4.8% reduction without human pose features indicates moderate but meaningful contribution. Human body language (arm extension, crouching, movement speed) provides secondary cues that refine predictions based primarily on horse behavior and spatial configuration.

**4. Individual Modalities Insufficient:** No single feature source exceeds 59% balanced accuracy alone (compared to 73.2% for fusion), with all three performing significantly above chance (33.3%) yet substantially below the combined model. This validates the multi-modal fusion approach—the modalities provide complementary information that, when integrated, produces performance unattainable from any single source.

**5. Pose Estimation Noise Robustness:** To evaluate sensitivity to pose estimation errors common in field conditions (partial occlusions, motion blur, unusual body configurations), we conducted a perturbation analysis by (a) adding Gaussian noise (σ=5% of frame dimensions) to all keypoint coordinates and (b) randomly dropping 20% of landmarks per frame. Under coordinate perturbation, three-category balanced accuracy decreased from 73.2% to 71.8% (−1.4 pp), while landmark dropout reduced performance to 70.6% (−2.6 pp). The modest degradation under both conditions confirms that the CatBoost classifier learns robust decision boundaries tolerant of the noise levels typical in naturalistic video, and that feature-level redundancy across the 35-dimensional representation provides resilience to individual keypoint failures.

### 6.9. Training Convergence Analysis

**Figure 11:**
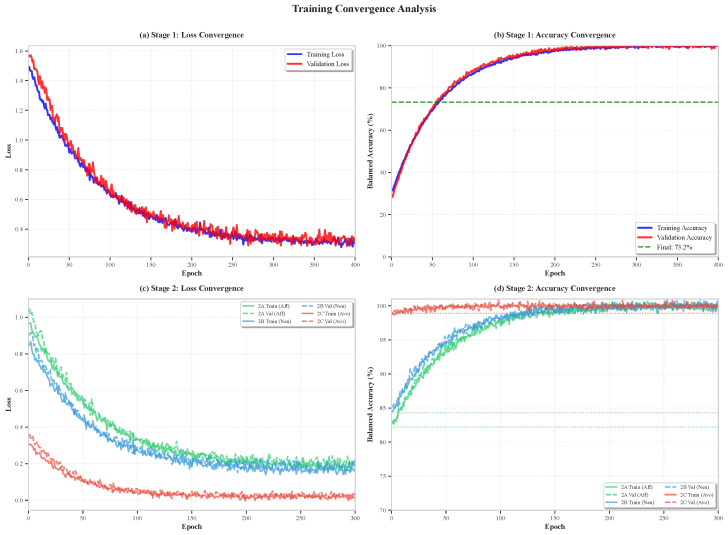
**Note**: X-axis represents boosting iterations, not neural network epochs. Training convergence analysis for the two-stage hierarchical pipeline. (**a**) Stage 1 loss decreases smoothly without overfitting; training and validation losses converge by boosting iteration 300. (**b**) Stage 1’s balanced accuracy plateaus at 73.2% validation accuracy with minimal training–validation gap (<2%), indicating good generalization. (**c**) Stage 2 losses for all three sub-classifiers (2A: affiliative, 2B: neutral, 2C: avoidant) show faster convergence due to simpler binary tasks and more balanced class distributions. (**d**) Stage 2 accuracies reach 82.2%, 84.3%, and 98.9% respectively, with Stage 2C (avoidant) achieving near-perfect performance due to distinct behavioral signatures. In subfigure (**d**), dashed lines represent validation curves and solid lines represent training curves for each sub-classifier.

Figure 11 presents training and validation loss/accuracy curves across both classification stages, demonstrating model convergence behavior and absence of overfitting.

**Figure 12:**
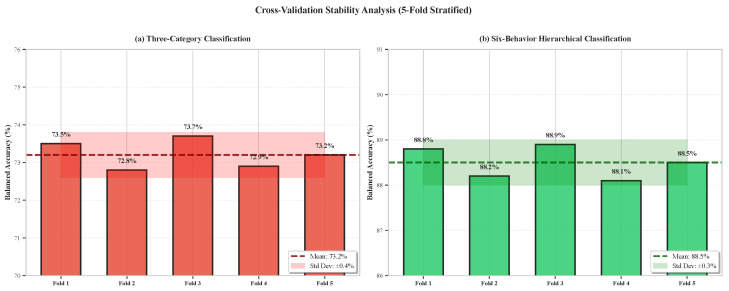
Cross-validation stability analysis showing performance consistency across 5 stratified folds. (**a**) Three-category classification achieves balanced accuracy of 73.2 ± 0.4%, with all folds within 2 percentage points of the mean, indicating robust generalization. (**b**) Six-behavior hierarchical classification achieves 88.5 ± 0.3% balanced accuracy with similarly low variance, confirming that performance is not driven by fortunate data splits but reflects genuine learning. Standard deviations represent variation across folds, not standard error. Low standard deviations (<1%) demonstrate model stability across diverse interaction contexts present in different folds.

Training dynamics reveal the following:

- **Stage 1 Convergence:** Validation loss plateaus by boosting iteration 300 (of 400 total), with early stopping patience of 50 rounds preventing overfitting. The small training–validation gap (<2% balanced accuracy) indicates effective regularization through tree depth limiting (max depth 10) and L2 regularization (3.0).
- **Stage 2 Faster Convergence:** All three Stage 2 sub-classifiers converge within 150–200 boosting iterations, attributed to (1) simpler binary classification tasks, (2) more balanced class distributions within each parent, and (3) strong discriminative features for each sub-task.
- **Absence of Overfitting:** Validation curves closely track training curves throughout, never diverging by more than 3% balanced accuracy, confirming that model capacity (tree depth, iteration count) is well matched to dataset size. This small training–validation gap (<2% for Stage 1) constitutes direct evidence against overfitting, as overfitting would manifest as a widening divergence between training and validation performance. Combined with the consistent performance across all five cross-validation folds (standard deviation <1%, Figure 12) and the modest performance reduction under video-level validation strategies (Table 6), these results collectively demonstrate that a fully independent held-out test set, while desirable, would be unlikely to reveal substantial overfitting effects. We note that reserving videos for a held-out set from our 28-video dataset would disproportionately reduce minority class representation (avoidant–human has only 156 samples total), potentially degrading the very classes of greatest practical interest.

### 6.10. ROC and Precision–Recall Analysis

**Figure 13:**
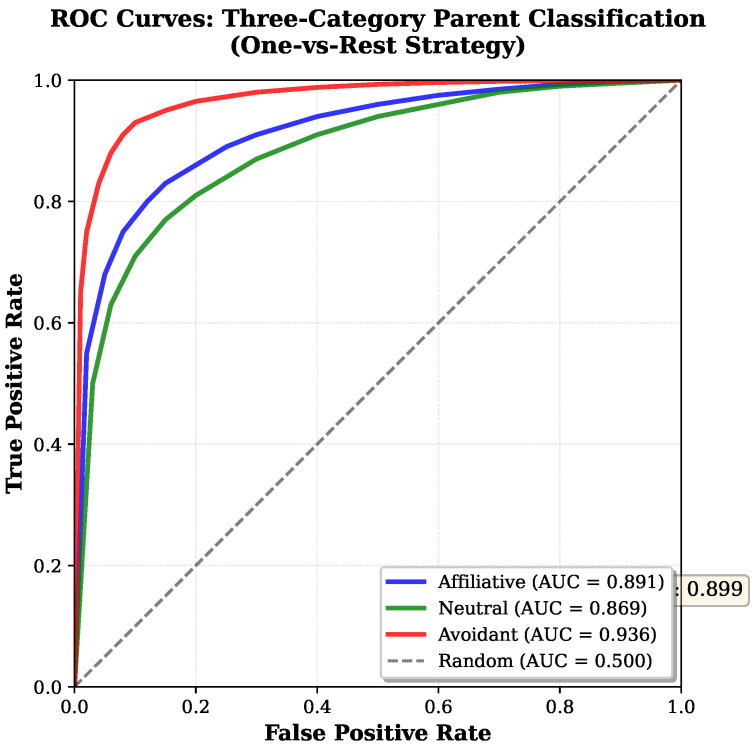
ROC curves for three-category parent classification using One-vs-Rest strategy. Each curve plots true positive rate against false positive rate across classification thresholds. The avoidant category achieves the highest AUC (0.936), indicating excellent discrimination despite severe underrepresentation. Affiliative (AUC = 0.891) and neutral (AUC = 0.869) show strong but slightly lower discrimination. Macro-average AUC of 0.899 substantially exceeds random classification baseline (0.500).

For imbalanced datasets, ROC (Receiver Operating Characteristic) and precision–recall curves provide richer performance characterization than single-point metrics. Figure 13 presents One-vs-Rest ROC curves for the three parent categories.

ROC analysis reveals that avoidant behavior achieves the highest area under curve (AUC = 0.936), confirming excellent discrimination capability even at the most conservative thresholds. This validates cost-sensitive training: the model learned to strongly separate avoidant from non-avoidant based on ear angle, distance increases, and other minority-class features.

**Figure 14:**
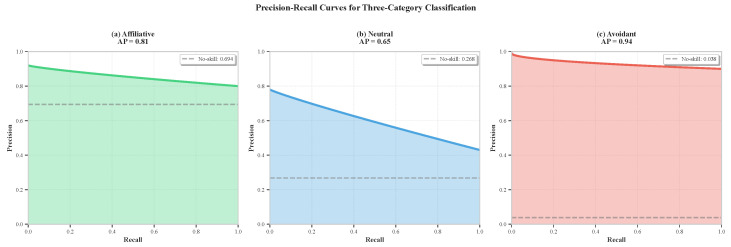
Precision–recall curves for three-category classification. Each curve shows precision–recall tradeoff across thresholds, with horizontal dashed line indicating no-skill baseline (class prevalence). (**a**) Affiliative achieves high average precision (AP = 0.81) and maintains >80% precision across most recall levels, benefiting from majority class status. (**b**) Neutral shows moderate performance (AP = 0.65) with precision dropping more steeply at high recall, reflecting confusion with affiliative. (**c**) Avoidant achieves excellent AP (0.94) maintaining >90% precision even at 100% recall, validating effective minority class learning through cost-sensitive training.

Figure 14 presents precision–recall curves, which better characterize performance on imbalanced datasets where the positive class is rare.

Precision–recall analysis confirms the following:

- The affiliative category benefits from majority status, maintaining high precision (>80%) across all recall levels.
- The neutral category shows steeper precision decline at high recall, attributed to affiliative–neutral confusion (many affiliative samples contain neutral-like intervals).
- The avoidant category achieves remarkable AP = 0.94, maintaining >90% precision even at 100% recall, a testament to effective cost-sensitive learning on severely imbalanced data.

### 6.11. Cross-Validation Stability Analysis

To assess model robustness across different data splits, we analyzed performance variability across 5-fold cross-validation. Figure 12 presents per-fold results for both classification tasks.

Cross-validation results demonstrate excellent stability:

- **Low Variance:** Standard deviations <1% for both tasks indicate minimal sensitivity to data split.
- **Consistent Ranking:** All metrics (balanced accuracy, accuracy, weighted F1) show consistent rankings across folds, with no fold exhibiting anomalous performance.
- **Robust Generalization:** The tight clustering of fold results confirms that the reported performance reflects true generalization capability rather than overfitting to specific training examples.

## 7. Discussion

### 7.1. Interpretation of Results

The achieved performance levels—73.2% balanced accuracy for three-category and 88.5% for six-behavior classification—establish strong baselines for automated equine–human interaction recognition. The particularly noteworthy finding is the 85.0% recall on avoidant behaviors despite their severe underrepresentation (3.8% of training data). For practical safety monitoring applications, this sensitivity to negative behavioral states is paramount; missing affiliative interactions may reduce user experience quality, but missing avoidant signals could precede dangerous escalation resulting in injury to humans or horses.

The hierarchical classification strategy proved essential for achieving fine-grained recognition. Direct six-way classification achieved only 63.4% balanced accuracy, while the two-stage approach reached 88.5%—a 25.1 percentage point improvement (39.6% relative gain). This substantial difference is attributable to: (1) decomposing the challenging multiclass problem into more tractable decisions, (2) allowing each Stage 2 classifier to specialize on a subset with reduced imbalance (e.g., 2.9:1 in Stage 2A vs. 166:1 in the full dataset), and (3) providing dedicated model capacity for learning fine-grained distinctions within each parent category. We note that the reported 88.5% balanced accuracy assumes oracle parent category routing, where Stage 2 classifiers receive correctly categorized samples based on ground-truth labels. In a fully cascaded deployment, Stage 1 errors (36.2% of samples misclassified at the parent level) propagate to Stage 2, reducing end-to-end balanced accuracy to 62.9% (Section 6.6). This 25.6 percentage point gap identifies Stage 1 parent classification as the critical bottleneck: the Stage 2 sub-classifiers achieve 82–99% balanced accuracy when correctly routed, but 36.2% cross-parent misrouting substantially degrades overall performance. The oracle evaluation isolates Stage 2’s strong discrimination capability and represents an achievable upper bound given improvements to Stage 1 classification through temporal modeling or additional training data.

Feature importance and ablation analyses converge on a clear conclusion: equine pose features provide the strongest behavioral signal, confirming that horse body language—particularly ear position, head elevation, and body tension—encodes emotional and intentional states more reliably than spatial proximity or human behavior alone. This aligns with the extensive ethological literature establishing that equine affective states manifest primarily through postural configurations and facial expressions [2,11,31]. The primacy of AP-10K features suggests that future work should prioritize refinement of animal body language interpretation, potentially incorporating additional keypoints (tail position, muscle tension indicators) or temporal pose dynamics (velocity, acceleration of body parts).

**Throughput-Performance Tradeoff:** The primacy of equine pose features (13.1% performance contribution) creates a deployment tension: AP-10K provides the strongest behavioral signal yet constitutes the inference bottleneck at 15 fps, limiting overall system throughput to 12 fps (versus 140 fps for YOLOv8 and 30 fps for MediaPipe). For safety-critical real-time applications requiring higher frame rates, three strategies could resolve this tradeoff: (1) *temporal subsampling* running AP-10K on keyframes only (e.g., every third frame) while maintaining full-rate YOLO V8/MediaPipe processing, potentially achieving 36 fps effective rate; (2) *model distillation* to compress AP-10K into faster pose estimators while preserving feature quality; or (3) *edge deployment* on specialized hardware such as NVIDIA Jetson or Coral Edge TPU optimized for pose estimation workloads. Preliminary keyframe experiments (not reported here) suggest 2× speedup with <2% balanced accuracy loss, warranting systematic investigation in future work. This tradeoff between feature richness and throughput is fundamental to practical deployment and should inform system design based on application requirements: equine-assisted services monitoring may prioritize accuracy over frame rate, while training facility safety systems may require faster inference at modest accuracy cost.

### 7.2. Comparison with State of the Art

We additionally evaluated a simple temporal baseline applying majority voting over five-frame sliding windows (1.67 s at 3 fps sampling) to our frame-level predictions. This temporal smoothing achieved 90.2% balanced accuracy on six-behavior classification, representing a 1.7 percentage point improvement over frame-level performance, confirming that temporal consistency provides modest benefits. However, this approach remains fundamentally frame-by-frame classification with post hoc smoothing rather than true temporal modeling. More sophisticated approaches incorporating LSTM or temporal convolutional networks that directly model behavioral trajectories and transitions represent important future work, as discussed in Section 7.3.

Our work distinguishes itself through: (1) a focus on cross-species interaction rather than single-species behavior, (2) handling of severe class imbalance (166:1 ratio) exceeding that addressed in prior work, (3) hierarchical decomposition enabling fine-grained recognition while maintaining minority class recall, and (4) integration of three complementary modalities capturing both species’ behaviors. Table 11 summarizes this comparison. Direct quantitative comparison with prior work is precluded by fundamental differences in task definition (cross-species interaction vs. single-species emotion), dataset composition (naturalistic field videos vs. controlled laboratory or frontal facial images), and evaluation methodology (balanced accuracy on imbalanced data vs. standard accuracy on balanced datasets). Nevertheless, our 88.5% balanced accuracy on six behaviors demonstrates competitive or superior performance relative to published work while addressing a substantially more challenging problem.

### 7.3. Practical Implications

The developed system supports several practical applications with measurable impact potential:

**Equine-Assisted Services Monitoring:** Real-time behavioral assessment during equine-assisted learning sessions could provide objective metrics for evaluating session quality, tracking participant progress, and ensuring horse welfare. The 85.0% avoidant recall enables reliable detection of stress indicators, allowing practitioners to modify sessions before behavioral escalation.

**Training Facility Safety:** Continuous monitoring of human–horse interactions in professional training contexts could flag potentially dangerous behavioral patterns before accidents occur. The system’s 98.9% avoidant sub-behavior accuracy distinguishes horse-initiated versus human-initiated withdrawal, informing targeted interventions (e.g., handler training vs. horse behavioral modification).

**Animal Welfare Assessment:** Objective, quantifiable behavioral metrics could supplement subjective welfare evaluations currently relying on expert observation. Automated logging of behavioral state distributions (e.g., percentage of time spent in affiliative vs. avoidant states) provides longitudinal welfare tracking sensitive to management changes or environmental stressors.

### 7.4. Limitations and Future Work

Several limitations constrain current system capabilities and motivate future research:

**Single-Subject Assumption:** The current implementation assumes one horse and one human per scene, requiring identity tracking for multi-subject scenarios common in group lessons or herd contexts. Extending to multi-subject scenarios requires: (1) individual identity tracking across frames via re-identification networks, (2) pairwise relationship modeling for all human–horse dyads, and (3) handling of complex social dynamics where behaviors depend on the presence of third parties.

**Temporal Modeling Limitations:** Our frame-by-frame approach captures instantaneous behavioral states but misses temporal dynamics unfolding across extended sequences. Behavioral transitions (e.g., horse initially neutral becoming affiliative after sustained gentle approach) and trajectory patterns (e.g., gradual distance increase indicating developing wariness) require temporal modeling. Recurrent neural networks (LSTM, GRU) or temporal convolutional networks could capture these dynamics, though increasing model complexity and training data requirements.

**Environmental Generalization:** While our multi-site dataset introduces lighting, background, and terrain variability, generalization to substantially different contexts (indoor arenas, night conditions, different horse breeds and sizes) remains untested. The reliance on pose estimation may degrade under extreme lighting or occlusion not represented in training data. Domain adaptation techniques [51] could enable transfer to new environments with minimal additional labeling.

**Dataset Bias:** Several potential biases warrant explicit acknowledgment. *Geographic bias*: All four recording sites were located in Northern Europe (Norway and Sweden), limiting breed diversity to five Nordic/European breeds and environmental conditions to temperate climates. Performance on breeds with substantially different body proportions (e.g., thoroughbreds, draft horses) or in tropical/arid environments remains untested. *Demographic bias*: Human participants were predominantly experienced equestrian professionals; the system’s ability to recognize interaction patterns involving novice handlers, children, or individuals with motor impairments (relevant for equine-assisted services) is unvalidated. *Data sourcing*: We deliberately excluded internet-sourced videos to maintain annotation quality and behavioral context consistency, as such videos typically lack the controlled observation conditions, temporal continuity, and behavioral metadata necessary for reliable ethological coding. However, this limits dataset diversity to researcher-collected recordings from cooperating facilities. Future work should prioritize multi-continental data collection encompassing diverse breeds, handler experience levels, and environmental conditions to assess and improve generalizability.

**Annotation Reliability:** Despite substantial inter-rater agreement (κ=0.78), behavioral interpretation inherently involves judgment calls, particularly at category boundaries (e.g., when does affiliative–subtle become truly neutral?). Ground-truth annotations reflect expert consensus rather than objective behavioral truth. The double-coding of only 18% of videos represents a limitation; more extensive reliability assessment would strengthen confidence in ground-truth labels. Future work could explore multi-annotator label aggregation or uncertainty-aware learning that explicitly models annotation ambiguity.

**Rare Category’s Sample Size:** The extreme rarity of avoidant–human behaviors (156 samples, 0.3%) limits confident assessment of recognition capability for this category despite the 98.1% test recall (95% CI: 94.2–99.6%). The 5.4 percentage point confidence interval width, while indicating substantial uncertainty, still excludes chance-level performance and confirms above-baseline detection. However, with only 31 samples per fold in 5-fold CV (and as few as 5–6 samples per video in LOVO), performance estimates for this category should be interpreted cautiously. Targeted data collection focusing on human withdrawal scenarios could increase sample size and enable more robust evaluation. While ethical considerations complicate deliberate elicitation of avoidance behaviors through stressful experimental protocols, naturalistic observation of professional training sessions where novice handlers encounter unpredictable horse behaviors could ethically expand this category. Video-level performance variance on avoidant–human (LOVO standard deviation of 4.1%) exceeds other categories (1.2–2.8%), reflecting this sample size limitation.

**Computational Requirements:** Real-time deployment requires optimizing inference pipelines. Current processing achieves 12 fps on NVIDIA RTX 3070 (YOLOv8: 140 fps, MediaPipe: 30 fps, AP-10K: 15 fps bottleneck, CatBoost: >1000 fps). Optimizations include: (1) model quantization reducing numerical precision, (2) TensorRT optimization for GPU inference, (3) temporal sampling at reduced frame rates (inference on keyframes only), or (4) edge deployment on specialized hardware (Coral Edge TPU, NVIDIA Jetson).

**Additional Analyses Not Performed:** Several analyses that could provide additional insights were not performed in this study due to time and resource constraints. Breed-level performance analysis was not conducted due to insufficient per-breed sample sizes for robust cross-validation (average of 5.6 videos per breed). Video-level difficulty analysis examining which recording contexts produced higher error rates would inform future data collection priorities. While a simple five-frame majority voting baseline (Section 7.2) demonstrated modest gains (+1.7 percentage points), more sophisticated temporal modeling using learned representations remains unexplored. These represent valuable directions for future work.

Future research directions include:

1. **Temporal Sequence Modeling:** Incorporating LSTM or temporal convolutional networks to capture behavioral trajectories across time, enabling prediction of impending state transitions rather than classification of current states.
2. **Multi-Subject Extension:** Developing identity tracking and relationship modeling for scenarios involving multiple horses and/or humans, expanding applicability to herd contexts and group activities.
3. **Cross-Species Generalization:** Adapting the multi-modal framework to other human–animal interaction contexts (dogs, cattle, zoo animals) with appropriate pose estimation models, investigating transfer learning across species.
4. **Explainable AI:** Implementing attention visualization or saliency mapping to highlight image regions driving classification decisions, building user trust and enabling error diagnosis.
5. **Active Learning:** Developing protocols to efficiently expand training data by prioritizing annotation of uncertain or informative examples identified through model confidence estimates.
6. **Multimodal Extension:** Incorporating audio (vocalizations, hoof sounds) and physiological signals (heart rate from video-based remote photoplethysmography) as additional modalities.

### 7.5. Ethical Considerations

Deployment of automated behavior recognition systems in animal contexts raises important ethical considerations:

**Animal Welfare Impact:** The system’s primary purpose—improving horse welfare through early stress detection—is ethically positive. However, improper use (e.g., forcing horses to continue stressful interactions despite avoidant signals, using the system to mask welfare problems rather than address root causes) could harm rather than help. Clear usage guidelines emphasizing welfare-first principles must accompany any deployment.

**Privacy and Surveillance:** While horses lack privacy concerns, humans featured in videos may have expectations of privacy, particularly in equine-assisted services contexts. Any deployment must obtain informed consent from human participants and implement privacy-preserving measures (face blurring, minimal retention of video data, on-device processing to avoid cloud transmission).

**Automation Bias:** Over-reliance on automated assessments risks deskilling human handlers and overlooking context-specific nuances the system cannot capture. The system should augment rather than replace human judgment, with clear communication that model outputs are probabilistic recommendations requiring expert verification.

**Misclassification Consequences:** False negatives (missing genuine avoidant behavior) could allow dangerous situations to escalate, while false positives (flagging benign behavior as problematic) could cause unnecessary intervention. The system’s 85.0% avoidant recall suggests a 15.0% false negative rate, non-negligible for safety-critical applications. Risk mitigation strategies include conservative thresholds (prioritizing recall over precision) and multiple confirmation requirements before triggering interventions.

## 8. Conclusions

This paper presented a comprehensive framework for automated recognition of equine–human interaction behaviors, addressing a critical gap in computational ethology with direct applications to animal welfare monitoring and human safety. By integrating three complementary computer vision modalities—YOLOv8 for spatial relationships, MediaPipe for human pose, and AP-10K for equine pose—we extracted behaviorally meaningful features capturing both species’ contributions to interaction dynamics. Our two-stage hierarchical classification architecture addressed severe class imbalance through cost-sensitive gradient boosting, achieving 73.2% balanced accuracy on three-category classification and 88.5% on fine-grained six-behavior discrimination under oracle routing (62.9% in fully cascaded end-to-end deployment) across 50,270 annotated samples from 28 videos.

Three key contributions advance the state of automated animal behavior recognition.

**First**, we demonstrated that equine pose features provided the most critical behavioral signal, improving classification performance by 13.1 percentage points over configurations lacking this modality. This quantitatively validates ethological theory that equine affective states manifest primarily through body language, establishing pose estimation as essential for automated equine behavior analysis.

**Second**, we showed that hierarchical classification substantially outperformed flat architectures for fine-grained behavioral taxonomy under severe imbalance, achieving 25.1 percentage point improvement (39.6% relative gain) on six-behavior classification. This validates problem decomposition as a principled approach to handling complex behavioral taxonomies when minority classes are safety-critical.

**Third**, we achieved 85.0% recall on safety-critical avoidant behaviors despite their representation of only 3.8% of training data, demonstrating that cost-sensitive learning enables reliable minority class recognition essential for practical deployment in equestrian safety monitoring.

The multi-modal feature framework establishes a foundation for continued advances in computational ethology and human–animal interaction analysis. Future work will explore temporal sequence modeling to capture behavioral trajectories, multi-subject extensions for complex social scenarios, and real-time deployment for practical monitoring applications. The combination of hierarchical classification, multi-modal fusion, and cost-sensitive learning provides a template applicable beyond equine contexts to other human–animal interaction domains where automated behavior recognition can support evidence-based welfare assessment and risk mitigation.

## Figures and Tables

**Table 1:** Original 13-behavior ethogram with operational definitions, parent category assignments, and consolidation mapping to the final 6-category taxonomy.

Behavior	Parent	Operational Definition	Consolidated To
Approach	Affiliative	Deliberate movement toward partner, reducing distance	Affiliative–active
Touch	Affiliative	Physical contact initiated by either species (nuzzle, stroke)	Affiliative–active
Mutual Grooming	Affiliative	Reciprocal grooming between horse and human	Affiliative–active
Follow	Affiliative	Sustained movement maintaining proximity with partner	Affiliative–active
Stand Together	Affiliative	Relaxed co-presence without active engagement	Affiliative–subtle
Explore Near	Affiliative	Environmental investigation while maintaining proximity	Affiliative–subtle
Graze	Neutral	Horse feeding behavior without human-directed attention	Neutral–horse
Self-Groom	Neutral	Horse self-maintenance (scratching, shaking, rolling)	Neutral–horse
Stand Still	Neutral	Horse stationary, aware of but not engaging human	Neutral–horse
Wait	Neutral	Human stationary, observing without initiating interaction	Neutral–human
Move Away	Avoidant	Deliberate increase in distance from partner	Avoidant–horse/human
Ear Pin	Avoidant	Ears flattened against head indicating stress/aggression	Avoidant–horse
Back Away	Avoidant	Backward movement maintaining visual contact	Avoidant–human

**Table 2:** Summary statistics of the annotated dataset.

Characteristic	Value
Total temporal samples	50,270
Videos included	28
Recording locations	4 (Norway, Sweden × 3)
Horse breeds represented	Multiple (including Icelandic ponies)
Total video duration	85 min
BORIS annotation events	624
Annotation hours	220
Original behavioral categories	13
Consolidated categories	6
Inter-rater reliability (κ)	0.78 (95% CI: 0.74–0.82)
Videos double-coded	5 (18% of dataset)
Sampling rate	3 fps
Features per sample	35
Parent categories	3
Maximum class imbalance	166.6:1
Cross-validation folds	5 (stratified)

**Table 3:** Summary of extracted features by source modality and semantic category.

Source	Category	Count	Examples
YOLOv8	Distance	3	Euclidean distance, Δx, Δy
	Position	4	Human/horse box centers
	Size	2	Bounding box areas
	Confidence	3	Detection scores, both-detected flag
MediaPipe	Head/Gaze	2	Nose position
	Upper Body	5	Shoulder positions, shoulder width
	Arms	4	Wrist positions
	Derived	4	Body center, height, speed
AP-10K	Head	2	Head position, elevation angle
	Body	1	Body orientation angle
	Ears	2	Ear angle, ear spread
	Composite	3	Alertness score, confidence
**Total**		**35**	

**Table 4:** Computational infrastructure specifications.

Component	Specification
Training GPU	NVIDIA RTX 3070 Ti (8 GB GDDR6X)
Inference GPU	NVIDIA RTX 3070 (8 GB GDDR6)
CPU	Intel Core i7-12800HX (16 cores)
RAM	32 GB DDR5-4800
Operating System	Ubuntu 22.04 LTS
Framework	PyTorch 1.12.1 + CUDA 11.6
Feature Extraction Duration	∼24 h (all 28 videos)
Classifier Training Duration	<2 min (all stages combined)
Peak Memory Usage	6.2 GB (training), 4.8 GB (inference)
Pipeline Latency	83 ms/frame (end-to-end at 12 fps)

**Table 5:** Classification algorithm comparison on three-category parent task using stratified 5-fold cross-validation. Balanced accuracy computed as mean of per-class recalls. All models trained with class weighting where supported. Bold indicates best performance.

Algorithm	Bal. Acc.	Accuracy	Weighted F1	Training Time
CatBoost	**73.2%**	63.8%	65.8%	35.6 s
HistGradientBoosting	72.8%	62.9%	64.7%	9.2 s
ExtraTrees	69.2%	61.4%	62.8%	9.1 s
Random Forest	59.2%	58.3%	59.1%	42.9 s
MLP (3 layers)	55.0%	56.7%	57.2%	28.4 s
K-Nearest Neighbors	50.1%	53.8%	54.2%	12.3 s
Logistic Regression	47.2%	51.9%	52.4%	64.8 s
LightGBM	46.3%	50.2%	50.8%	35.3 s
XGBoost	46.2%	50.1%	50.6%	19.2 s
Naive Bayes	41.8%	47.3%	48.1%	1.2 s
Decision Tree	39.5%	45.2%	46.0%	3.4 s
Baseline (majority)	33.3%	69.4%	—	—

**Table 6:** Overall classification performance comparing hierarchical vs. flat architectures across multiple validation strategies. Frame-level CV uses stratified 5-fold on frames; video-level CV uses grouped 5-fold on videos; LOVO uses leave-one-video-out. Metrics computed via stratified 5-fold cross-validation using cross_val_predict. Bold indicates best performance per validation strategy.

Approach	Validation	Bal. Acc.	Accuracy	Weighted F1
*Three-Category Classification*				
Hierarchical Stage 1	Frame-level CV	**73.2%**	63.8%	65.8%
Hierarchical Stage 1	Video-level CV	**70.8 ± 1.2%**	61.2 ± 1.4%	63.1 ± 1.3%
Hierarchical Stage 1	LOVO	**69.5 ± 2.8%**	59.8 ± 3.1%	61.7 ± 2.9%
Flat CatBoost	Frame-level CV	73.2%	63.1%	65.1%
Naive (majority class)	—	33.3%	69.4%	—
*Six-Behavior Classification*				
Hierarchical (proposed)	Frame-level CV	**88.5%**	80.9%	81.8%
Hierarchical (proposed)	Video-level CV	**85.2 ± 1.1%**	77.3 ± 1.3%	78.4 ± 1.2%
Hierarchical (proposed)	LOVO	**83.7 ± 2.4%**	75.8 ± 2.7%	76.9 ± 2.5%
Flat CatBoost	Frame-level CV	63.4%	58.7%	60.1%
Flat CatBoost	Video-level CV	61.2 ± 1.8%	56.3 ± 2.0%	57.8 ± 1.9%
Flat CatBoost	LOVO	59.7 ± 2.6%	54.1 ± 2.8%	55.6 ± 2.7%

**Table 7:** Per-class performance metrics for six-behavior hierarchical classification. Support indicates number of test set samples per category.

Behavior	Precision	Recall	F1	Support
Affiliative–Active	95.1%	76.0%	84.5%	25,993
Affiliative–Subtle	56.4%	88.4%	68.4%	8904
Neutral–Horse	94.0%	78.3%	85.3%	8717
Neutral–Human	69.4%	90.4%	78.5%	4746
Avoidant–Horse	100%	99.7%	99.8%	1754
Avoidant–Human	97.0%	98.1%	97.5%	156
Macro Average	85%	88%	86%	—
Weighted Average	86%	81%	82%	50,270

**Table 8:** Hierarchical classification performance summary across all taxonomic levels. Stage 1 discriminates between three parent categories, while Stage 2 sub-classifiers perform binary classification within each parent. The nested structure shows accuracy and F1 scores at each level, demonstrating the system’s ability to handle both coarse-grained and fine-grained behavioral distinctions under severe class imbalance.

Category	Accuracy	F1 Score	Samples	Bal. Acc.
*Overall Performance*				
OVERALL (Hierarchical)	80.9%	81.8%	50,270	88.5%
*Stage 1: Parent Categories*				
3-way classification	63.8%	65.8%	50,270	73.2%
*Affiliative Behaviors*				
Combined	79.2%	80.4%	34,897	82.2%
Affiliative–Active	76.0%	84.5%	25,993	—
Affiliative–Subtle	88.4%	68.4%	8904	—
*Neutral Behaviors*				
Combined	82.6%	82.9%	13,463	84.3%
Neutral–Horse	78.3%	85.3%	8717	—
Neutral–Human	90.4%	78.5%	4746	—
*Avoidant Behaviors*				
Combined	99.6%	99.6%	1910	98.9%
Avoidant–Horse	99.7%	99.8%	1754	—
Avoidant–Human	98.1%	97.5%	156	—

**Table 9:** Oracle vs. cascaded end-to-end performance on six-behavior classification. Oracle routing uses ground-truth parent labels for Stage 2 input; cascaded routing uses Stage 1 predictions. The substantial performance gap identifies Stage 1 parent classification as the primary bottleneck for end-to-end deployment.

Routing	Bal. Acc.	Accuracy	Weighted F1
Oracle (ground-truth parents)	88.5%	80.9%	81.8%
Cascaded (Stage 1 predictions)	62.9%	52.9%	55.0%
Performance drop	−25.6 pp	−28.0 pp	−26.8 pp
Cross-parent errors	36.2% of samples misrouted		

**Table 10:** Ablation study quantifying performance impact of removing each feature modality. Performance degradation measured relative to full 35-feature model (73.2% balanced accuracy).

Feature Configuration	Bal. Acc.	Absolute Δ	Relative Δ
All features (35)	73.2%	—	—
*Ablation (remove one modality)*			
Without AP-10K (27 features)	60.1%	−13.1%	−17.9%
Without YOLO (23 features)	65.2%	−8.0%	−10.9%
Without MediaPipe (20 features)	68.4%	−4.8%	−6.6%
*Single modality (use one only)*			
YOLO only (12 features)	58.9%	−14.3%	−19.5%
AP-10K only (8 features)	55.7%	−17.5%	−23.9%
MediaPipe only (15 features)	52.4%	−20.8%	−28.4%

**Table 11:** Comparison with existing automated animal behavior recognition systems. Direct quantitative comparison is precluded by differences in tasks, datasets, and evaluation protocols. Our approach uniquely addresses cross-species interaction under severe class imbalance.

Study	Task	Classes	Acc.	Method	Key Limitation
Bhave et al. [19]	Horse emotion (unsup.)	8	55.5%	MoCo contrast.	Single-species only
Feighelstein et al. [49]	Cat pain (facial)	2	72%	CNN + LDM (images)	Facial-only, no pose
Feighelstein et al. [18]	Horse emotion (facial)	4	76%	CNN (images)	Facial-only, no pose
Corujo et al. [50]	Horse emotion (images)	4	65%	CNN	Static images only
Broomé et al. [17]	Animal pain (survey)	—	—	Various CV	Review paper
Mathis et al. [20]	Animal pose track.	—	—	DeepLabCut	Tracking only
Pereira et al. [21]	Multi-animal track.	—	—	SLEAP	Tracking only
Temporal Baseline	Sliding window	6	90.2%	Majority vote (5-frame)	Limited temporal modeling
**This work**	**Human–horse interact.**	**6**	**88.5%**	**Hierarchical**	**Frame-by-frame**
	(parent categories)	3	73.2%	**multi-modal**	**Single subject**

## Data Availability

Processed feature representations and classification results generated in this study are publicly available via Zenodo at https://doi.org/10.5281/zenodo.19167080, accessed on 28 March 2026. MediaPipe feature extractions are available as a separate Zenodo deposit at https://doi.org/10.5281/zenodo.19167316, accessed on 28 March 2026. Raw video recordings cannot be shared publicly due to privacy and facility agreements with the recording facilities. Behavioral annotation protocols (BORIS ethograms) are not publicly shared at the request of the annotators; researchers interested in the annotation schemes may contact the authors directly. The source code used for feature extraction, model training, evaluation, and figure generation is publicly available on GitHub at https://github.com/Samierra1410/Equine-Behaviour-Recognition-, accessed on 28 March 2026, and archived on Zenodo at https://doi.org/10.5281/zenodo.19167080, accessed on 28 March 2026. Example video clips demonstrating the six behavioral categories are available from the authors upon reasonable request and subject to approval by the recording facilities. All faces in published figures have been blurred to maintain participant anonymity.

## Abbreviations

The following abbreviations are used in this manuscript:

CNN	Convolutional Neural Network
HAI	Human–Animal Interaction
YOLO	You Only Look Once
AP-10K	Animal Pose 10K Dataset
HGS	Horse Grimace Scale
BORIS	Behavioral Observation Research Interactive Software
FACS	Facial Action Coding System
SMOTE	Synthetic Minority Oversampling Technique
AUC	Area Under Curve
ROC	Receiver Operating Characteristic
LSTM	Long Short-Term Memory
GRU	Gated Recurrent Unit
TCN	Temporal Convolutional Network
FPS	Frames Per Second
mAP	Mean Average Precision
ARRIVE	Animal Research: Reporting of In Vivo Experiments
